# Identification of procathepsin L (pCTS-L)–neutralizing monoclonal antibodies to treat potentially lethal sepsis

**DOI:** 10.1126/sciadv.adf4313

**Published:** 2023-02-03

**Authors:** Cassie Shu Zhu, Xiaoling Qiang, Weiqiang Chen, Jianhua Li, Xiqian Lan, Huan Yang, Jonathan Gong, Lance Becker, Ping Wang, Kevin J. Tracey, Haichao Wang

**Affiliations:** ^1^ The Feinstein Institutes for Medical Research, Northwell Health, 350 Community Drive, Manhasset, NY 11030, USA.; ^2^ Donald and Barbara Zucker School of Medicine at Hofstra/Northwell, 500 Hofstra Blvd., Hempstead, NY 11549, USA.

## Abstract

Antibody-based strategies have been attempted to antagonize early cytokines of sepsis, but not yet been tried to target inducible late-acting mediators. Here, we report that the expression and secretion of procathepsin-L (pCTS-L) was induced by serum amyloid A (SAA) in innate immune cells, contributing to its late and systemic accumulation in experimental and clinical sepsis. Recombinant pCTS-L induced interleukin-6 (IL-6), IL-8, GRO-α/KC, GRO-β/MIP-2, and MCP-1 release in innate immune cells and moderately correlated with blood concentrations of these cytokines/chemokines in clinical sepsis. Mechanistically, pCTS-L interacted with Toll-like receptor 4 (TLR4) and the receptor for advanced glycation end products (RAGE) to induce cytokines/chemokines. Pharmacological suppression of pCTS-L with neutralizing polyclonal and monoclonal antibodies attenuated pCTS-L–mediated inflammation by impairing its interaction with TLR4 and RAGE receptors, and consequently rescued animals from lethal sepsis. Our findings have suggested a possibility of developing antibody strategies to prevent dysregulated immune responses mediated by late-acting cytokines.

## Teaser

An antibody strategy is developed to attenuate pCTS-L-mediated dysregulated inflammation in sepsis.

## INTRODUCTION

Microbial infections and resultant sepsis syndromes are the most common causes of death in intensive care units, accounting for approximately 20% of total deaths worldwide (*1*). Its pathogenesis remains poorly understood but is partly attributable to dysregulated innate immune responses (e.g., hyperinflammation and immunosuppression) to lethal infections (*2*). To mount efficient inflammatory responses, innate immune cells use various pattern recognition receptors [PRRs; e.g., Toll-like receptor 4 (TLR4)] (*3*) to recognize distinct classes of molecules shared by related microbes known as “pathogen-associated molecular patterns” [PAMPs; e.g., bacterial endotoxins and lipopolysaccharide (LPS)]. For instance, upon detecting minute amount of endotoxins by an LPS-binding protein (LBP) (*4*), a co-receptor cluster of differentiation 14 (CD14) (*5*) delivers it to the high-affinity cell-surface PRR, TLR4 (*3*), thereby triggering the immediate release of “early” cytokines such as tumor necrosis factor (TNF) (*6*), interleukin-1β (IL-1β) (*7*), and interferon-γ (IFN-γ) (*8*).

However, if excessive amounts of LPS are internalized via CD14/TLR4-mediated endocytosis or bacterial outer membrane vesicles (*9*), it induces oligomerization and activation of cytoplasmic Casp-11/4/5 receptor (*10*), resulting in dysregulated pyroptosis and consequent leakage of late-acting damage-associated molecular patterns (DAMPs) such as high mobility group box 1 (HMGB1) (*11*) and sequestosome-1 (SQSTM1) (*12*). When HMGB1 is secreted by innate immune cells in relatively low amounts, it binds high-affinity TLR4 receptor to augment inflammation during an early stage of sepsis (*13*). However, when HMGB1 is passively released by pyroptotic cells in overwhelmingly higher quantities, it could also bind to other low-affinity receptors, such as the receptor for advanced glycation end product (RAGE), thereby inducing immune tolerance (*14*), pyroptosis (*15*, *16*), and immunosuppression (*17*) that may adversely compromise the host’s ability to eradicate microbial infections (*18*). Consequently, HMGB1 has been characterized as a late-acting DAMP and mediator of lethal sepsis with a relatively wider therapeutic window than early proinflammatory cytokines (*11*, *13*, *19*).

In parallel, early cytokines (e.g., TNF, IL-1β, and IFN-γ) also induce proinflammatory mediators such as serum amyloid A (SAA) in hepatocytes (*20*). Upon secretion, extracellular SAA uses TLR4 (*21*) and RAGE (*22*) to induce hemichannels (e.g., connexin 43 and pannexin 1) (*23*, *24*) and secretory phospholipase A_2_ (e.g., sPLA_2_-IIE/V) (*25*), thereby triggering HMGB1 release and serving as a mediator of lethal sepsis (*26*). However, it was previously unknown whether SAA also induces other “late-acting” cytokines that can be therapeutically targeted in a delayed regimen.

Cathepsin L (CTS-L) is a papain-like lysosomal enzyme responsible for degrading endocytosed proteins to generate immunogenic antigens for adaptive immunities. Although human and murine proCTS-L (pCTS-L) share nearly 86% of sequence homology, they exhibit only 59% homology to the counterpart pCTS-L of a distantly related liver fluke parasite (*27*). Unlike other papain enzymes, CTS-L is inducible in some malignantly transformed tumor cells by various growth factors, and the leader-less precursor, pCTS-L, can be secreted extracellularly (*28*) to facilitate tumor invasion and metastasis (*29*). Even in nontransformed cells, some inflammatory (e.g., LPS, IFN-γ, or IL-6) (*30*, *31*) and noxious stimuli [e.g., alcohol, cigarette smoking, and ultraviolet (UV) irradiation] (*32*–*34*) similarly stimulate the expression and secretion of pCTS-L in innate immune cells (*30*, *33*) or nonimmune cells such as hepatocytes (*32*), dermal fibroblasts (*34*), and synovial fibroblasts (*31*). However, it was previously unknown (i) whether an intermediate mediator, SAA, similarly induces pCTS-L expression and secretion and (ii) whether pCTS-L can be therapeutically targeted in a delayed fashion. Here, we provided compelling evidence to support (i) a previously unidentified role for extracellular pCTS-L in the pathogenesis of lethal sepsis and (ii) a promising therapeutic potential of pCTS-L–neutralizing monoclonal antibodies (mAbs) in preclinical model of sepsis.

## RESULTS

### Identification of pCTS-L as an SAA-inducible and secretory protein

**Fig. 1.**
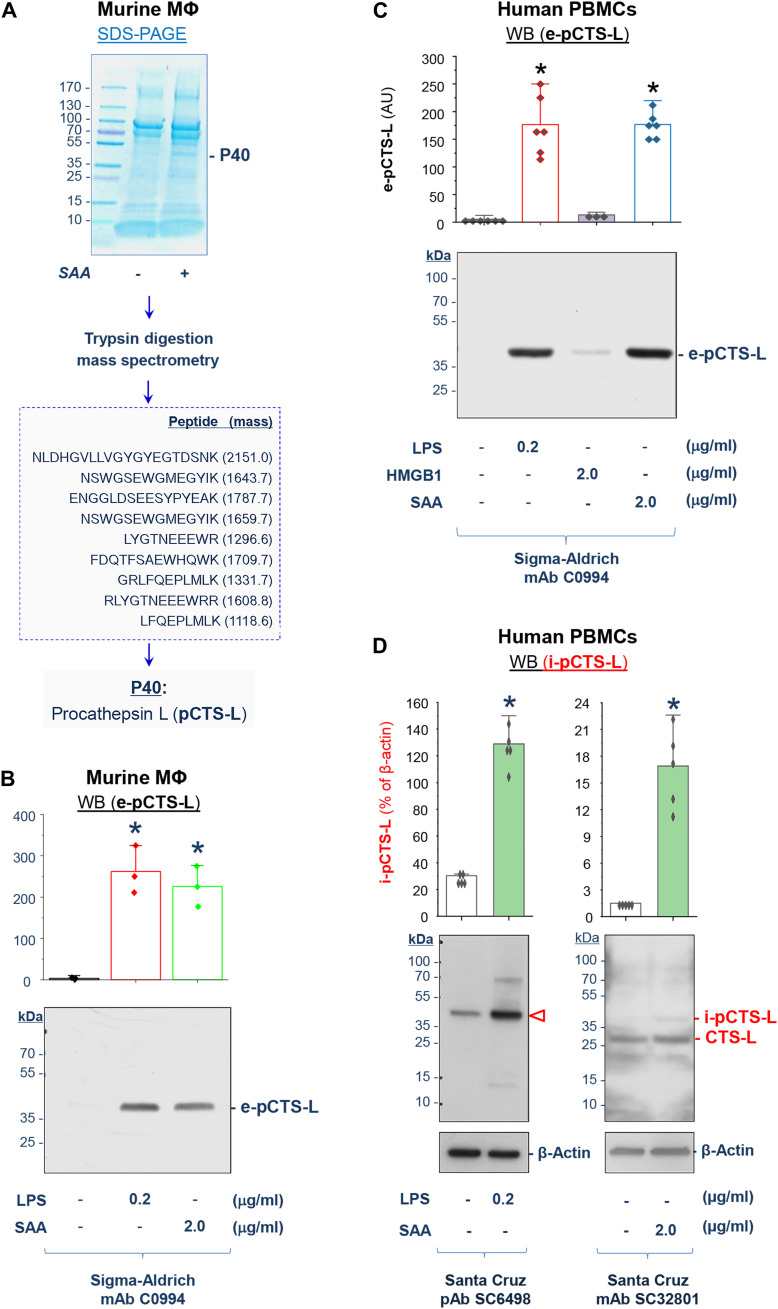
Identification of pCTS-L as an inducible and secretory protein from activated innate immune cells. (**A** and **B**) Characterization of pCTS-L as a secretory protein in LPS- and SAA-stimulated murine macrophages (MΦ). Murine macrophage-like RAW 264.7 cells were stimulated with SAA or LPS for 16 hours, and extracellular proteins in the macrophage-conditioned culture medium were resolved by SDS–polyacrylamide gel electrophoresis (SDS-PAGE). A 40-kDa SAA-inducible secretory protein (P40) was identified as pCTS-L by mass spectrometry (A) and confirmed by Western blotting (B) using a specific mAb. Bar graph represents quantitation of pCTS-L band intensities of three independent Western blots in arbitrary units (AU; *n* = 3 biological replicates). *P < 0.05 versus negative control (“− LPS − SAA”) by one-way analysis of variance (ANOVA) test. (**C** and **D**) Induction and secretion of pCTS-L in LPS- and SAA-stimulated human PBMCs. Human PBMCs were stimulated with LPS, HMGB1, or SAA for 16 hours, and levels of pCTS-L in the cell-conditioned medium [e-pCTS-L (C)] or whole-cell lysate [i-pCTS-L (D)] were determined by Western blotting using different antibodies. Sample loading was normalized by equal volume of culture medium conditioned by equivalent number of cells for extracellular proteins (C) or by β-actin for intracellular proteins (D). Bar graph represents quantitation of pCTS-L band intensities of five independent experiments in AU (*n* = 5 biological replicates) or % of β-actin. *P < 0.05 versus “− LPS − SAA − HMGB1” or “− LPS − SAA” by ANOVA test.

To search for late-acting mediators of sepsis, we characterized SAA-inducible proteins in murine macrophage-conditioned culture medium. Prolonged stimulation with SAA resulted in a marked elevation of the extracellular content of a 40-kDa protein (“P40”; Fig. 1A), which was identified as murine pCTS-L by in-gel trypsin digestion and mass spectrometry (MS) (Fig. 1A). To verify its identity, the culture medium conditioned by SAA- or LPS-stimulated macrophages was immunoblotted with a pCTS-L–specific mAb, which confirmed a marked pCTS-L release in response to SAA or LPS stimulation (Fig. 1B). In addition, we stimulated human primary peripheral blood mononuclear cells (PBMCs) with LPS, SAA, or HMGB1 for 16 hours and measured pCTS-L content in both PBMC-conditioned medium (“e-pCTS-L”; Fig. 1C and fig. S1A) and PBMC lysates (“i-pCTS-L”; Fig. 1D and fig. S1B) using different antibodies. The amounts of both intra- and extracellular pCTS-L were similarly elevated by LPS and SAA, but not by HMGB1 (Fig. 1, C and D, and fig. S1, A and B), suggesting that both microbial PAMPs and host proinflammatory mediators induced pCTS-L expression and secretion in innate immune cells.

### Up-regulation and systemic accumulation of pCTS-L in experimental sepsis

**Fig. 2.**
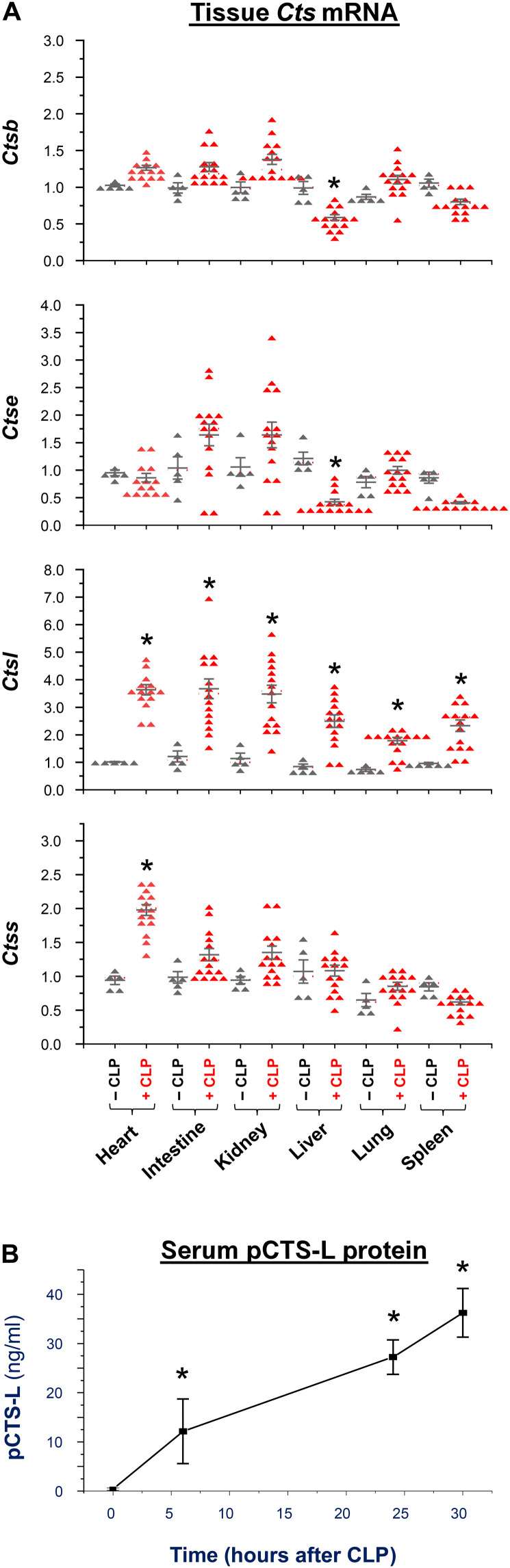
Blood pCTS-L protein concentrations were time dependently elevated in experimental sepsis. (**A**) Up-regulation of *Ctsl* mRNA in experimental sepsis. Male and female Balb/C mice were sacrificed at 24 hours after CLP to harvest various tissues to isolate total mRNAs for real-time RT-PCR analysis of *Ctsb*, *Ctse*, *Ctsl*, and *Ctss* mRNA expression (expressed as AU) with reference to a housekeeping gene, glyceraldehyde-3-phosphate dehydrogenase (GAPDH). *P < 0.05 versus “− CLP” by one-way ANOVA. (**B**) Blood pCTS-L levels were time dependently increased in experimental sepsis. Male Balb/C mice were sacrificed at various time points after CLP surgery to harvest blood samples for measuring serum pCTS-L contents by Western blotting with reference to highly purified recombinant pCTS-L at various dilutions. *N* = 5 mice (biological replicates). *P < 0.05 versus − CLP by one-way ANOVA.

To confirm its up-regulation in vivo, we used real-time reverse transcription polymerase chain reaction (RT-PCR) to survey the mRNA expression profiles of several *Cts* genes including *Ctsb*, *Ctse*, *Ctsl*, and *Ctss* in septic animals (Fig. 2A). In line with a possible role of CTS-S in murine and human pancreatitis (*35*), the expression of *Ctss* mRNA was selectively elevated in the heart of septic mice at 24 hours after cecal ligation and puncture (CLP) (Fig. 2A). In a sharp contrast, the expression of *Ctsl* mRNA was uniformly elevated in the heart, intestine, kidney, liver, lung, and spleen (Fig. 2A), suggesting that *Ctsl* was predominantly up-regulated in experimental sepsis. These findings were consistent with a previous report that *Ctsl* was up-regulated by threefold in the skeletal muscle as early as 2 days after *Escherichia coli* infection (*36*). To assess its kinetics of systemic accumulation, we measured circulating pCTS-L protein concentrations at several time points after CLP. Circulating pCTS-L was not detected in normal healthy animals, but time dependently elevated in septic animals, approaching plateau at 24 to 32 hours after CLP (Fig. 2B), a time period when some septic animals started to succumb to death. It suggested a relatively late and systemic pCTS-L accumulation in a preclinical model of sepsis.

### Systemic accumulation of pCTS-L and other surrogate biomarkers in clinical sepsis

**Fig. 3.**
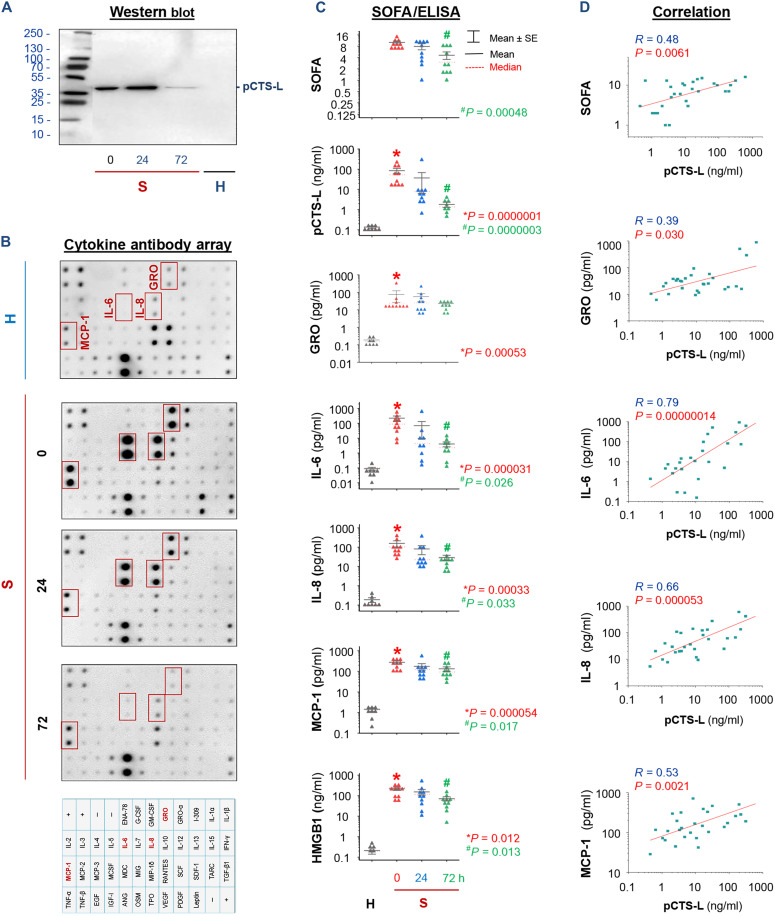
Blood pCTS-L levels were elevated in septic patients and moderately correlated with surrogate markers of experimental and clinical sepsis. (**A**) Western blotting of plasma pCTS-L in a representative normal healthy control (“H”) and a septic patient (“S”) at three time points after diagnosis. (**B**) Cytokine antibody array analysis of 42 cytokines and chemokines in a representative normal healthy control (H) and a septic patient at three time points after the clinical diagnosis. (**C**) Parallel changes of plasma pCTS-L levels with SOFA scores, as well as surrogate markers of sepsis. *n* = 8 to 10 biological replicates. *P value versus normal controls (“N”) and ^#^*P* value versus septic patient at time 0 (within 24 hours of sepsis diagnosis) by nonparametric Kruskal-Wallis ANOVA test. (**D**) Correlation between plasma pCTS-L levels and SOFA scores, as well as surrogate markers of sepsis. The Spearman rank correlation coefficient test was used to evaluate associations between two variables that exhibit nonnormal distribution.

To evaluate its clinical relevance, we used various immunoassays to characterize the dynamic changes of circulating pCTS-L in parallel with several surrogate markers of clinical sepsis. Although pCTS-L was not detected in the plasma of normal healthy controls (“H”; Fig. 3, A and C, and fig. S2, A and B), it was significantly elevated in age- and sex-matched septic patients at 0 to 72 hours after their clinical diagnosis (Fig. 3, A and B; fig. S2B; and table S2) and paralleled with the systemic accumulation of a late-acting DAMP, HMGB1 (Fig. 3C). In patients with clinical sepsis, the plasma pCTS-L contents also moderately correlated with corresponding sequential organ failure assessment (SOFA) scores (Fig. 3D) and the blood concentrations of several cytokines (IL-6) and chemokines (GRO, IL-8, and MCP-1; Fig. 3D) previously characterized as surrogate markers of experimental (*37*, *38*) and clinical sepsis (*39*). These findings have confirmed a sustainably late and systemic accumulation of pCTS-L in preclinical and clinical settings.

### Requirement of TLR4 and RAGE receptors for pCTS-L–induced dysregulated inflammation

**Fig. 4.**
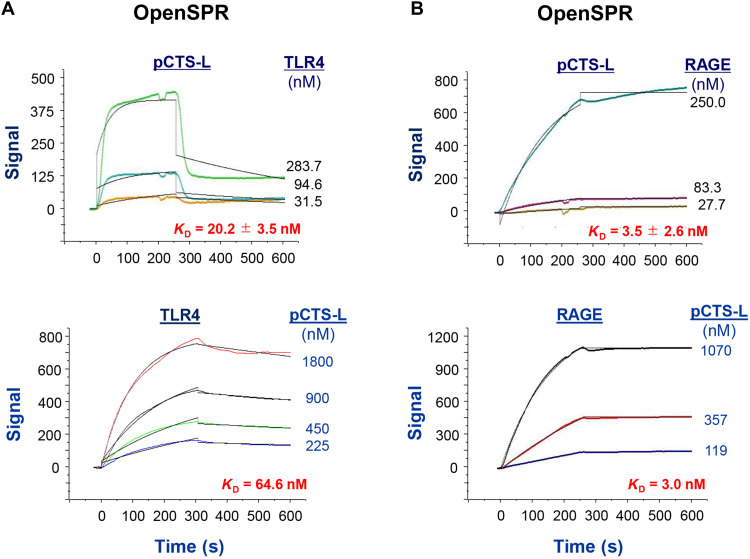
pCTS-L dose dependently interacted with TLR4 and RAGE receptor. Recombinant human pCTS-L (**A** and **B**, top), human TLR4 [residues 1 to 631 (A, bottom)], or the extracellular domain of RAGE [residues 1 to 344 (B, bottom)] was immobilized on the NTA sensor chip, and various analytes (e.g., TLR4, RAGE, or pCTS-L) were respectively applied as analyte at different concentrations to estimate the equilibrium dissociation constant *K*_d_. Representative *K*_d_ or mean ± SEM of eight independent experiments was shown (*n* = 8 technical replicates).

In light of the TLR4 dependence of a distantly related liver fluke pCTS-L (fig. S3A) in activating dendritic cells (*27*), we generated recombinant human and murine pCTS-L proteins (fig. S3B) to verify their possible interaction with TLR4 and other receptors using the Open Surface Plasmon Resonance (OpenSPR). When highly purified human pCTS-L was immobilized onto a sensor chip and human TLR4 or RAGE was respectively applied as analytes at various concentrations, pCTS-L exhibited high affinities to both TLR4 [with an estimated equilibrium dissociation constant (*K*_d_) = 20.2 ± 3.5 nM; Fig. 4A, top] and RAGE (*K*_d_ = 3.5 ± 2.6 nM; Fig. 4B, top). Conversely, when TLR4 or RAGE receptor was respectively conjugated to a sensor chip and pCTS-L ligand was applied as analyte, pCTS-L exhibited a similarly strong interaction with TLR4 (*K*_d_ = 64.4 nM; Fig. 4A, bottom) and RAGE (*K*_d_ = 3.0 nM; Fig. 4B, bottom), suggesting a possibility that pCTS-L might elicit dysregulated inflammation through these PRRs.

**Fig. 5.**
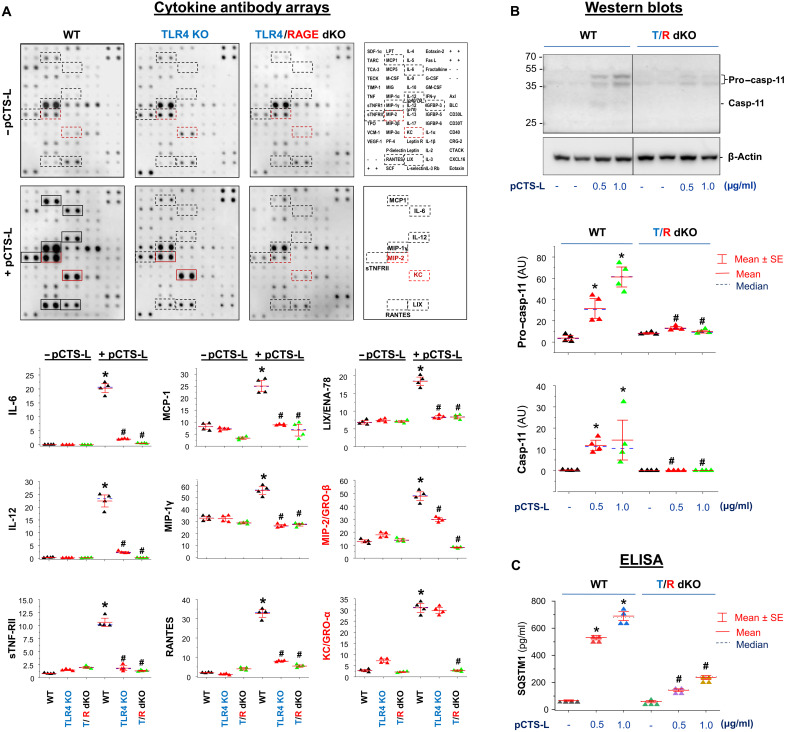
Critical roles of TLR4 and RAGE in pCTS-induced secretion of cytokines and chemokines. (**A**) Disruption of both TLR4 and RAGE completely abrogated pCTS-L–induced production of cytokines and chemokines. Thioglycollate-elicited primary macrophages were isolated from WT C57BL/6 or mutant mice deficient in either TLR4 alone (TLR4 KO) or both TLR4 and RAGE [TLR4/RAGE double KO (T/R dKO)]. Following stimulation with pCTS-L (0.5 μg/ml) for 16 hours, the extracellular levels of cytokines and chemokines were determined by cytokine antibody arrays. *n* = 4 mice per group (biological replicates). *P < 0.05 versus negative control (“− pCTS-L”) of respective genotype and ^#^*P* < 0.05 versus positive control (“+ pCTS-L”) of WT. (**B** and **C**) Disruption of both TLR4 and RAGE abrogated pCTS-L–induced Casp-11 expression and SQSTM1 release. *n* = 4 mice per group (biological replicates). *P < 0.05 versus negative control (− pCTS-L) of respective genotype and ^#^*P* < 0.05 versus positive control (+ pCTS-L) of WT with respective treatment regimens (0.5 or 1.0 μg/ml).

To test this possibility, we first determined whether genetic disruption of TLR4 and RAGE expression impaired pCTS-L–induced dysregulated inflammation. The disruption of TLR4 alone markedly reduced the pCTS-L–induced secretion of most cytokines (e.g., IL-6 and IL-12) and chemokines (e.g., RANTES, MCP-1, MIP-1γ, and LIX) except for MIP-2/GRO-β and KC/GRO-α (Fig. 5A), two neutrophilic surrogate markers of experimental sepsis (*37*). However, the double knockout (KO) of both TLR4 and RAGE resulted in a complete abolishment of all pCTS-L–induced cytokines and chemokines that included KC/GRO-α and MIP-2/GRO-β (Fig. 5A and fig. S4). Similarly, intraperitoneal administration of pCTS-L induced systemic accumulation of granulocyte colony-stimulating factor (G-CSF), soluble tumor necrosis factor receptor I (sTNF-RI), and several chemokines (e.g., BLC and MIP-1γ) in wild-type (WT) (fig. S5) but not in mutant mice deficient in TLR4 and RAGE (fig. S5), confirming an important role for both TLR4 and RAGE in pCTS-L–mediated dysregulated inflammation in vivo.

Excessive inflammation is often accompanied by a parallel up-regulation and activation of Casp-11/4/5, which triggers dysregulated inflammasome activation, pyroptosis, and passive DAMP release (*12*). pCTS-L dose dependently induced pro–Casp-11 expression and Casp-11 maturation in peritoneal macrophages derived from WT but not from TLR4/RAGE-deficient mice (Fig. 5B). Similarly, pCTS-L dose dependently induced the release of SQSTM1 from WT (Fig. 5C) but not TLR4/RAGE-deficient macrophages (Fig. 5C). Collectively, these findings support an important role for TLR4 and RAGE in pCTS-L–mediated dysregulated inflammation as well as in Casp-11–associated dysregulated pyroptosis and immunosuppression.

### Requirement of TLR4 and RAGE receptor for pCTS-L–induced tissue injury

**Fig. 6.**
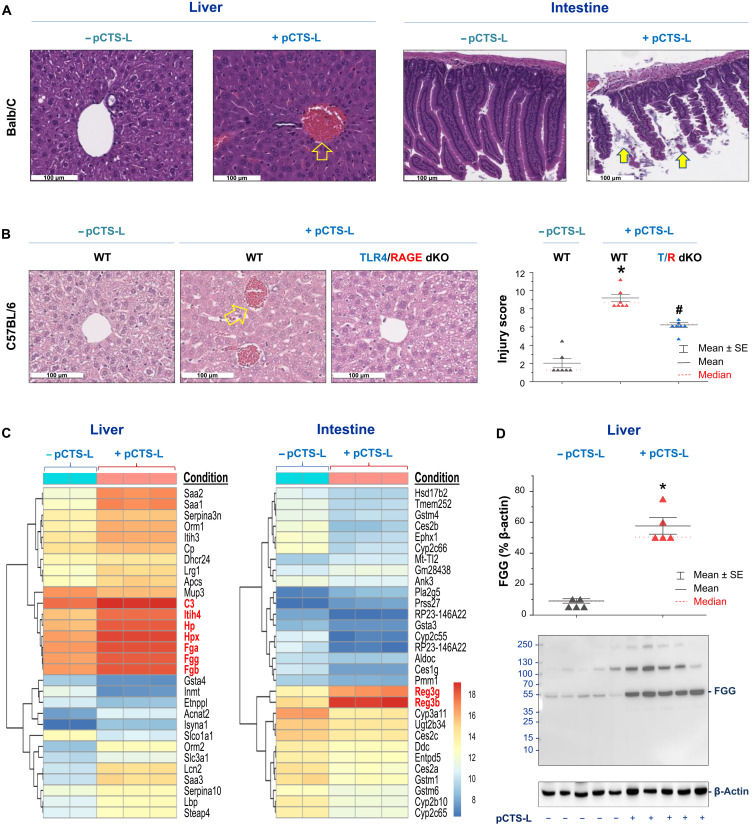
Intraperitoneal administration of recombinant pCTS-L caused more severe pathological changes in the WT than in mutant mice deficient in TLR4 and RAGE. (**A** and **B**) pCTS-L induced more severe liver injury in WT Balb/C or C57BL/6 than in mutant mice deficient in TLR4 and RAGE. Recombinant murine pCTS-L was intraperitoneally given to WT Balb/C [20 mg/kg (A)] or C57BL/6 mice [40 mg/kg (B)] or mutant C57BL/6 mice deficient in TLR4 and RAGE [40 mg/kg (B)], and intestine and/or liver tissues were harvested 24 hours later for hematoxylin and eosin staining. *n* = 7 samples per group (biological replicates). *P < 0.05 versus negative control (− pCTS-L) and ^#^*P* < 0.05 versus positive control (+ pCTS-L) of WT. (**C**) Heatmap of top 30 genes differentially expressed in the pCTS-L–challenged Balb/C mice. A biclustering heatmap was used to visualize the expression profile of the top 30 differentially expressed genes that were sorted by their adjusted *P* value and log_2_ fold changes. Genes with an adjusted *P* value of <0.05 and absolute log_2_ fold change of >2 were defined as differentially expressed. Each row represents a gene, and each column represents one sample from each animal. (**D**) pCTS-L markedly elevated hepatic fibrinogen-γ (FGG) protein content. Recombinant pCTS-L was intraperitoneally administered into Balb/C mice at a pathological dose (20 mg/kg), and hepatic tissues were harvested 20 hours later to measure hepatic FGG content by Western blotting. *n* = 5 mice per group (biological replicates). *P < 0.05 versus normal control (− pCTS-L).

To elucidate the role of pCTS-L in sepsis, we intraperitoneally injected mice with recombinant pCTS-L at suprapathological doses and found that pCTS-L caused marked injuries to the liver (i.e., the increase in hepatocellular necrosis, cytoplasmic vacuolization, and sinusoidal congestion; Fig. 6, A and B) and intestine (i.e., the loss of villi; Fig. 6A). Notably, the pCTS-L–induced tissue injuries were more rigorous in WT C57BL/6 mice as compared with mutant C57BL/6 mice deficient in both TLR4 and RAGE (Fig. 6B), supporting an important role for these PRRs in pCTS-L–mediated tissue injuries.

To further elucidate the mechanisms underlying pCTS-L–mediated pathogenesis, normal healthy Balb/C mice were intraperitoneally administered with recombinant pCTS-L, and various tissues were harvested to characterize the expression profile of a full catalog of transcripts by RNA sequencing (RNA-seq). Administration of pCTS-L markedly up-regulated several genes involved in the acute-phase response (e.g., SAA1/2/3, Hp, and Hpx) and coagulation (e.g., Fga, Fgb, and Fgg) in the liver (Fig. 6C) and bacterial killing (e.g., Reg3b and Reg3g) in the intestine (Fig. 6C). For instance, administration of pCTS-L induced a marked increase in hepatic mRNA expression (Fig. 6C) and protein content (Fig. 6D) of fibrinogen-γ (FGG), a substrate for thrombin-catalyzed production of fibrin, which can aggregate to form three-dimensional structural network capable of binding platelets and trapping blood cells to form thrombus (*40*). However, the possible roles of other altered genes in the pathogenesis of pCTS-L–mediated tissue injury remain an exciting subject of future studies.

### Genetic deletion of *Ctsl* conferred protection against sepsis-induced tissue injury

**Fig. 7.**
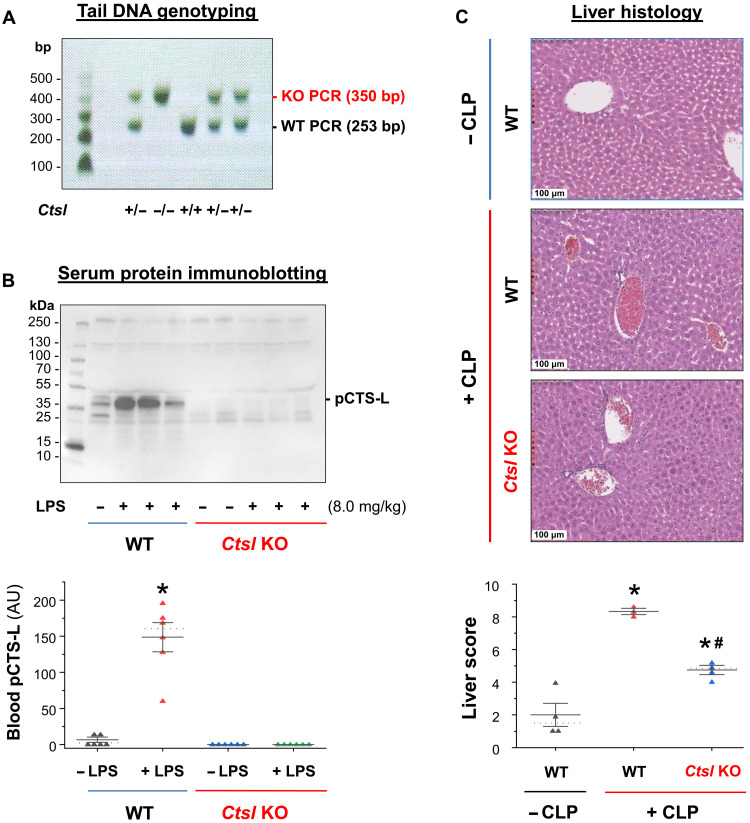
Disruption of Ctsl expression attenuated sepsis-induced liver injury. (**A** and **B**) Characterization of *Ctsl* KO mice by genotyping and immunoblotting of tail and serum samples. Genotyping analysis of WT [+/+, 253 base pairs (bp)], heterozygous (+/−, 253 and 350 bp), and homozygous (−/−, 350 bp) *Ctsl* gene by PCR amplification of genomic DNA extracted from mouse tail tissue using specific primers. Western blotting analysis of blood pCTS-L concentrations in WT and homozygous *Ctsl* KO mice at 24 hours after intraperitoneal administration of bacterial endotoxin (LPS, 8.0 mg/kg) using rabbit pAbs raised against murine pCTS-L. *n* = 6 mice per group (biological replicates). *P < 0.05 versus negative control (“− LPS”). (**C**) WT or *Ctsl* KO mice were subjected to CLP surgery, and liver was harvested at 24 hours after CLP for hematoxylin and eosin staining and histological analysis. Liver injury scores were expressed as means ± SEM of three to four animals per group. *n* = 3 to 4 mice per group (biological replicates). *P < 0.05 versus negative control (− CLP) and ^#^*P* < 0.05 versus positive control (+ CLP) of WT.

To confirm the pathogenic role of pCTS-L in sepsis, we first attempted the genetic gene KO approach by obtaining a few breeding pairs of heterozygous *Ctsl*^+/−^ KO mice. Unfortunately, female *Ctsl*^−/−^ KO mice were found infertile, because CTS-L was still needed for the normal degradation of the follicle wall to release mature oocytes during the process of ovulation (*41*). Similarly, male *Ctsl*^−/−^ KO mice might also suffer from poor reproductivity, because mutant mice expressing inactive CTS-L were also found abnormal in spermatogenesis (*42*). Therefore, we bred heterozygous *Ctsl*^+/−^ mice to produce a limited number of genetic background-, age-, and sex-matched WT littermates (*Ctsl*^+/+^) and pCTS-L–deficient (*Ctsl*^−/−^) mice. The genotypes of WT littermates and *Ctsl* KO mice were confirmed by genotyping (Fig. 7A) and immunoblotting (Fig. 7B) of tail and serum samples, respectively. Consistent with previous findings that genetic deletion of *Ctsl* led to an attenuation of pancreatitis (*43*), atherosclerosis (*44*), intimal hyperplasia (*45*), and antigen-induced arthritis (*46*), we found that genetic disruption of pCTS-L similarly lessened CLP-induced injury to the liver (Fig. 7C), confirming a pathogenic role of pCTS-L in lethal sepsis.

### pCTS-L–neutralizing pAbs conferred protection against lethal sepsis

To confirm its extracellular role in sepsis, we generated pCTS-L–specific polyclonal antibodies (pAbs) in rabbits (fig. S6) and tested their pharmacological effects on the lethal outcomes of sepsis. The epitope profiles of anti–pCTS-L rabbit serum were determined by dot blotting with 24 synthetic peptides corresponding to different region of murine or human pCTS-L proteins (figs. S6, A and B, and S7A), which revealed the presence of pAbs recognizing two homologous peptides corresponding to residues 175 to 193 (“P12”) and residues 194 to 214 (“P13”) of murine and human pCTS-L (figs. S6B and S7A). Anti-murine pCTS-L total immunoglobulin Gs (IgGs) (pAbs) conferred a dose-dependent and significant protection against lethal sepsis in both male and female mice when the first dose was given at 2 hours after CLP (Fig. 7B, top left). Anti-murine pCTS-L rabbit serum was subjected to protein A affinity chromatography to harvest total IgGs (“pAbs”; fig. S7A), which was then subjected to pCTS-L antigen affinity chromatography to isolate antigen-binding IgGs (A-IgGs) (fig. S7A). In a sharp contrast to the non–pCTS-L–binding control IgGs (C-IgGs), pCTS-L antigen affinity-purified IgGs (A-IgGs) completely abrogated the pCTS-L–induced production of cytokines and chemokines (fig. S7B), which included the TLR4-dependent IL-6, MIP-1γ, LIX, RANTES, and MCP-1 (Fig. 5B) as well as the RAGE-dependent KC/GRO-α and MIP-2/GRO-β (Fig. 5B and fig. S4). Thus, our findings suggested that anti–pCTS-L pAbs conferred protection against lethal sepsis partly by attenuating pCTS-L–induced dysregulated inflammation orchestrated by both TLR4 and RAGE receptors.

### Generation of mAbs against murine and human pCTS-L

**Fig. 8.**
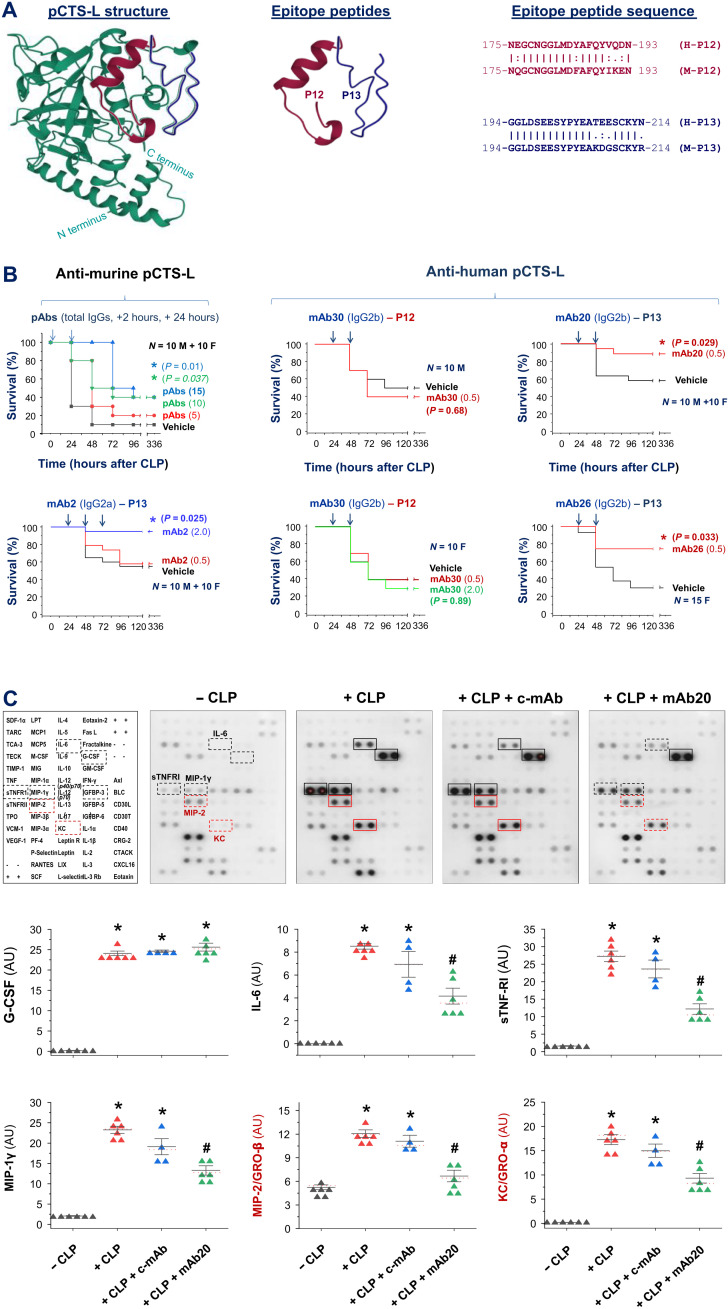
pCTS-L–specific pAbs and mAbs conferred a significant protection against lethal sepsis. (**A**) Conformation of human pCTS-L and two mAb-targeting peptides. The position of two epitope peptides (P12 and P13) was marked. (**B**) pAbs and mAbs raised against murine or human pCTS-L rescued mice from lethal sepsis. Male (M) or female (F) Balb/C mice were subjected to CLP, pAbs or mAbs against two different epitopes (P12 or P13) of human and murine pCTS-L were given intraperitoneally at indicated doses and time points, and animal survival rates were monitored for 2 weeks to ensure no later death. *n* = 10 to 20 mice per group (biological replicates). *P < 0.05 versus saline control group. (**C**) A protective mAb significantly attenuated sepsis-induced systemic inflammation. Balb/C mice were subjected to CLP sepsis, and a control mAb (c-mAb; 0.5 mg/kg) or protective mAb (mAb20, 0.5 mg/kg) was given twice at 2 and 20 hours after CLP. At 24 hours after CLP, animals were sacrificed to harvest blood and measure various cytokines and chemokines and expressed as AU. *n* = 4 to 6 mice per group (biological replicates). *P < 0.05 versus negative control (− CLP) and ^#^*P* < 0.05 versus positive control (+ CLP) group.

To develop potential therapies for human sepsis, we generated mAbs against both murine and human pCTS-L and explored their therapeutic potential by administering the first dose of mAb to septic animals in a delayed fashion—starting at 24 hours after CLP, a time point when circulating pCTS-L approached plateau concentrations and some septic animals started to succumb to death. Most of the mAbs cross-reacted with two homologous peptides corresponding to residues 175 to 193 (P12) or residues 194 to 214 (P13) of murine and human pCTS-L (Fig. 8A and fig. S8, A and B). Notably, mAbs cross-reacting with P12 peptide (e.g., mAb30) did not significantly affect animal survival when given at a wide range of doses (0.5 to 2.0 mg/kg; Fig. 8B, middle panels). In a sharp contrast, three mAbs recognizing P13 peptides of both human and murine pCTS-L effectively rescued animals from lethal sepsis even when the first dose was given at 24 hours after the onset of sepsis (Fig. 8B, bottom left and right panels). Consistently, the protective mAb significantly attenuated sepsis-induced systemic accumulation of IL-6, sTNF-RI, and several chemokines (e.g., MIP-1γ, MIP-2/GRO-β, and KC/GRO-α; Fig. 8C), suggesting that these beneficial antibodies confer protection against lethal sepsis possibly by attenuating sepsis-induced dysregulated inflammation.

### Anti–pCTS-L mAb attenuated pCTS-L–induced inflammation in human PBMCs

**Fig. 9.**
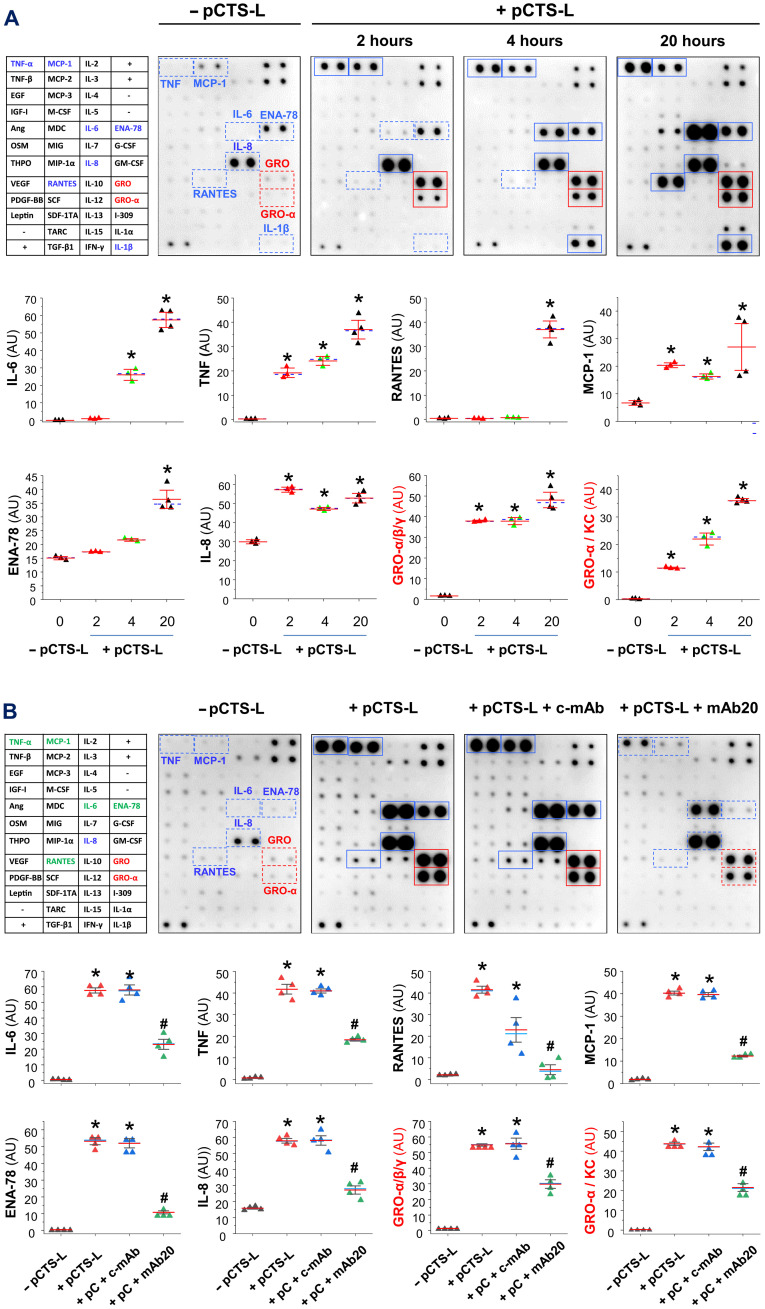
Protective mAb attenuated the pCTS-L–induced cytokines and chemokines in human PBMCs. (**A** and **B**) A protective mAb effectively inhibited pCTS-L–induced cytokines and chemokines in human PBMCs. Human PBMCs were stimulated with recombinant human pCTS-L for different time periods [2, 4, 16, and 20 hours (A)], and extracellular concentrations of cytokines and chemokines were determined by cytokine antibody arrays and expressed as AU. Note that pCTS-L induced the release of several cytokines and chemokines, which were effectively inhibited by a protective monoclonal antibody [mAb20 (B)] but not irrelevant control [c-mAb (B)]. *n* = 4 samples per group (biological replicates). *P < 0.05 versus negative control (− pCTS-L).

To further elucidate the protective mechanisms of pCTS-L–specific mAbs, we examined their effects on pCTS-L–induced production of cytokines/chemokines in primary human PBMCs. Recombinant pCTS-L time dependently induced several cytokines (e.g., IL-6 and TNF) and chemokines (e.g., RANTES, MCP-1, ENA-78/LIX, IL-8, GRO-α/KC, and GRO-α/β/γ) in human PBMCs (Fig. 9A and fig. S9). These inflammatory activities were not likely due to contaminating bacterial endotoxins, because extensive extractions of recombinant pCTS-L with Triton-X114 effectively reduced endotoxin content to <0.01 U/μg protein. Likewise, the endotoxin-free recombinant pCTS-L produced in eukaryotic [human embryonic kidney (HEK) 293] cells similarly induced these cytokines/chemokines in human PBMCs (fig. S9). Moreover, the pCTS-L–induced production of these cytokines/chemokines was markedly suppressed by the co-addition of a protective anti–pCTS-L mAb (mAb20; Fig. 9B) and not affected by an irrelevant control mAb (c-mAb; Fig. 9B). Collectively, these findings suggest that anti–pCTS-L mAbs confer protection against sepsis possibly by neutralizing the proinflammatory activities of extracellular pCTS-L.

**Fig. 10.**
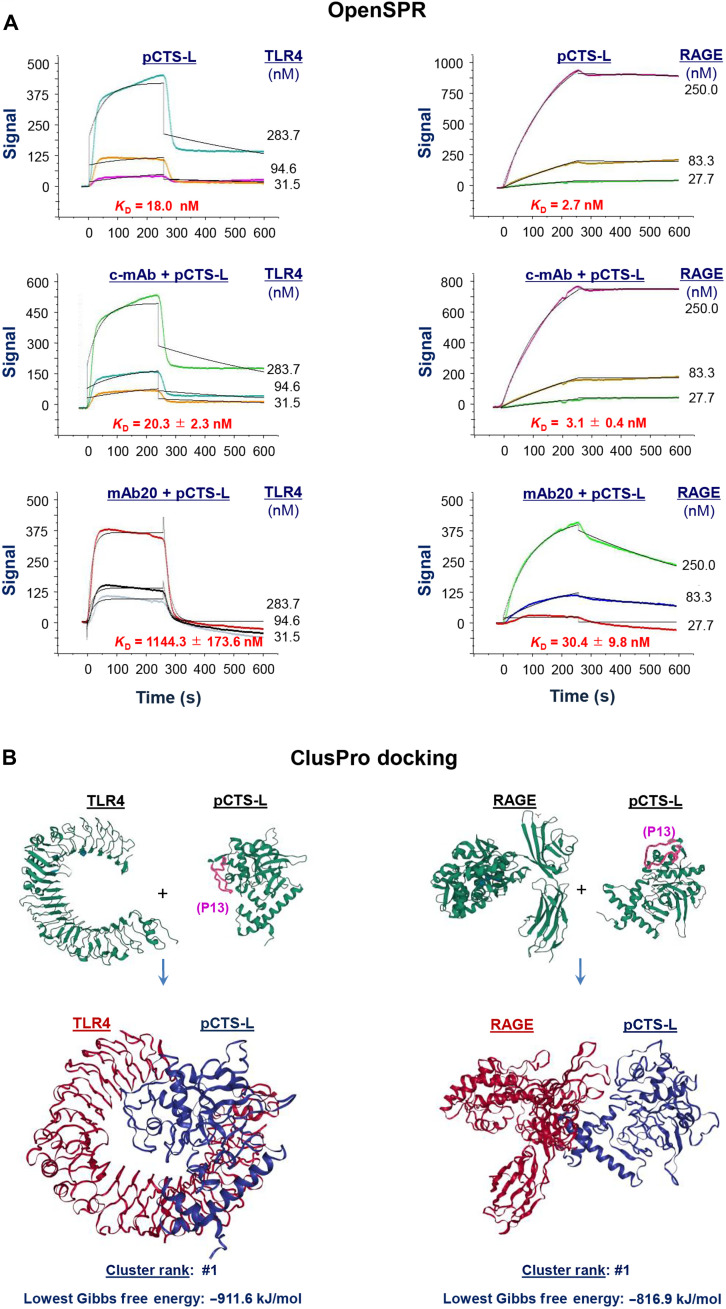
A protective anti–pCTS-L mAb markedly inhibited pCTS-L interaction with TLR4 and RAGE receptors. (**A**) Effect of a protective mAb on pCTS-L–receptor interactions. Recombinant pCTS-L was immobilized on the NTA sensor chip, and an irrelevant c-mAb or an anti–pCTS-L protective mAb (mAb20) was separately preincubated with pCTS-L–conjugated sensor chip before subsequent application of recombinant TLR4 (left) or RAGE (right) at various concentrations to estimate *K*_d_. Key experiments were performed three times (*n* = 3 technical replicates) to ensure reproducibility. (**B**) Protein-protein docking of pCTS-L/receptor complexes. We used the ClusPro Web Server to predict possible structures of pCTS-L/receptor complexes that exhibited the least Gibbs free energy. The epitope sequence for protective mAbs was marked on pCTS-L (as P13 in purple) to illustrate its relative position within the pCTS-L/receptor complexes. Note that the epitope sequence (P13) for protective mAbs was sequestered into the hydrophobic crevices of TLR4 (left) but positioned sideways in close proximity to the V-domain of RAGE (right).

To further understand the neutralizing mechanism of these protective anti–pCTS-L mAbs, we examined their effects on pCTS-L interaction with TLR4 and RAGE receptors. An irrelevant c-mAb did not affect pCTS-L interaction with neither TLR4 nor RAGE when it was preincubated with the pCTS-L–conjugated sensor chip even at extremely high concentrations (e.g., 1200 nM; Fig. 10A). In a sharp contrast, a protective anti–pCTS-L mAb (mAb20) markedly reduced pCTS-L’s affinities to both receptors, as manifested by an almost 55-fold (from 20.3 ± 2.3 nM to 1144.3 ± 173.6 nM) and 10-fold (from 3.1 ± 0.4 nM to 30.4 ± 9.8 nM) increase in the *K*_d_ for TLR4 and RAGE, respectively (Fig. 10A). To gain insight into receptor-ligand interactions, we used ClusPro protein-protein docking to find pCTS-L/receptor complex configurations that exhibited the minimal Gibbs free energy (Fig. 10B and fig. S10). In the most stable pCTS-L/receptor complexes, the epitope sequence (P13) for protective mAbs was sequestered into the hydrophobic crevices of TLR4 (Fig. 10B, left panels, and movie S1) but positioned sideways in close proximity to the V-domain of RAGE (Fig. 10B, right panels, and movie S2). The possibly different physical hindrance arisen from the engagement of P13-binding mAb20 might underlie its divergent inhibition of pCTS-L interaction with TLR4 (by 55-fold; Fig. 10A) and RAGE (by 10-fold; Fig. 10A). It suggests that protective anti–pCTS-L mAbs attenuate pCTS-L–induced inflammation possibly by inhibiting its interaction with these putative PRRs.

## DISCUSSION

Lacking effective therapies for sepsis other than adjunctive use of antibiotics, fluid resuscitation, and supportive care (*47*), it is still urgent to search for other late-acting pathogenic mediators that offer wider therapeutic opportunities. In the present study, we demonstrated that a liver-derived proinflammatory mediator (*26*), SAA, induced the expression and secretion of pCTS-L in both murine macrophages and human PBMCs (fig. S11), contributing to its sustainably late and systemic accumulation in experimental and clinical sepsis. Our findings further extended previous observations that pCTS-L was induced not only by growth factors in malignant tumor cells (*28*) but also by inflammatory (e.g., LPS, IFN-γ, or IL-6) (*30*, *31*) and noxious (e.g., alcohol, cigarette smoking, and UV irradiation) stimuli (*32*–*34*) in innate immune cells (*30*, *33*) or nonimmune cells such as hepatocytes (*32*), dermal fibroblasts (*34*), and synovial fibroblasts (*31*). Moreover, our current findings of pCTS-L up-regulation and systemic accumulation in experimental and clinical sepsis were consistent with previous observations of pCTS-L induction in murine model of endotoxemia (*48*).

Consistent with a previous report that a distantly related liver fluke pCTS-L signals via TLR4 to activate dendritic cells (*27*), we further demonstrated that human and murine pCTS-L activated murine macrophages and human PBMCs through both TLR4- and RAGE-dependent signaling pathways (fig. S11). First, SPR analysis revealed a high affinity for pCTS-L both to TLR4 and RAGE regardless whether the ligand or respective receptors were conjugated to a sensor chip before application of relevant analytes at various concentrations. Second, the disruption of both TLR4 and RAGE completely abrogated pCTS-L–induced release of all cytokines (IL-6 and IL-12) and chemokines (e.g., RANTES, MCP-1, MIP-1γ, LIX/ENA-78, KC/GRO-α, and MIP-2/GRO-β). Third, pharmacological inhibition of pCTS-L interaction with TLR4 and RAGE with neutralizing mAbs simultaneously attenuated the pCTS-L–induced production of both TLR4-dependent (e.g., ENA-78/LIX, IL-8, RANTES, and MCP-1) and RAGE-dependent chemokines (e.g., GRO-α/KC and GRO-β/MIP-2). Last, genetic KO of TLR4 and RAGE rendered animals resistant to pCTS-L–induced inflammation, further confirming the important role of TLR4 and RAGE in pCTS-L–mediated dysregulated inflammation in vivo.

Notably, some of the pCTS-L–inducible cytokines (e.g., IL-6) and chemokines (e.g., IL-8, MCP-1, GRO-α/KC, and GRO-β/MIP-2) have been characterized as surrogate markers of experimental (*37*) and clinical sepsis (*39*), because their blood levels are markedly higher in septic animals approaching the state of moribund than those remaining in the state of nonmoribund (*38*). Furthermore, the blood levels of these cytokines/chemokines moderately correlated with septic patients’ clinical SOFA scores and blood pCTS-L concentrations, suggesting that a sustainably late and systemic pCTS-L accumulation may contribute to the pathogenesis of lethal sepsis. First, pCTS-L triggered dysregulated inflammation by inducing various cytokines (e.g., TNF and IL-6) and chemokines (e.g., MCP-1, IL-8, MCP-1, GRO-α/KC, and GRO-β/MIP-2) in innate immune cells (fig. S11). Second, pCTS-L might also cause dysregulated immunosuppression by activating Casp-11/4/5, pyroptosis (fig. S11), and passive release of pathogenic DAMPs (e.g., HMGB1 and SQSTM1) (*11*, *12*, *49*). Third, intraperitoneal administration of pCTS-L caused severe tissue injuries in normal healthy animals, whereas genetic disruption of *Ctsl* expression rendered animals resistant to CLP-induced tissue injury. Last, pCTS-L–neutralizing pAbs and mAbs conferred a significant protection against lethal sepsis possibly by attenuating pCTS-L–mediated dysregulated inflammation. This newly uncovered role of extracellular pCTS-L in sepsis further expanded its pathogenic involvement in other inflammatory diseases such as pancreatitis (*43*), atherosclerosis (*44*), renal disease (*50*), vascular intimal hyperplasia (*45*), arthritis (*46*), and colitis (*51*).

Although gene KO strategy has been routinely used to verify important roles of various signaling molecules in diseases, cautions should be exercised when using this genetic approach to evaluate extracellular roles of inflammatory mediators. For instance, despite the well-established pathogenic role of HMGB1 in infection- and injury-elicited inflammation (*13*), the disruption of HMGB1 expression adversely renders animals more susceptible to both infectious (*52*) or injurious insults (*53*), indicating distinct roles of intracellular and extracellular HMGB1 in health and diseases (*54*). We thus developed pAbs against murine pCTS-L and used these pharmacological agents to further elucidate its extracellular role in sepsis. Inspired by our findings that pCTS-L–neutralizing polyclonal IgGs conferred a significant protection against sepsis, we used the traditional hybridoma technology to generate a panel of therapeutic mAbs. Specifically, we immunized mice with both murine or human pCTS-L and attempted to produce mAbs capable of binding both murine and human pCTS-L to (i) first evaluate their therapeutic efficacy in murine model of experimental sepsis and (ii) then develop humanized antibodies for future clinical treatment of human sepsis. Furthermore, we sought to develop mAbs with potential neutralizing activities to impair the harmful interaction of pCTS-L with its PRRs, thereby blocking pCTS-L–induced dysregulated inflammation (fig. S11). Consequently, we have obtained a panel of mAbs (2H8A2, 20D5H6, and 26C7C9) that cross-reacted with both murine and human pCTS-L to inhibit its interaction with TLR4 and RAGE receptors, thereby rescuing mice from lethal sepsis even when the first dose was given in a delayed fashion.

There are a number of limitations to the present study: (i) We have not yet obtained sufficient number of critically ill patients who died of severe sepsis, so we do not know whether blood pCTS-L concentrations could be used as a surrogate marker or predictor of lethal outcomes of sepsis. (ii) We do not know whether anti–pCTS-L mAbs are protective in other infectious diseases such as coronavirus disease 2019 (COVID-19), although a single-cell meta-analysis of severe acute respiratory syndrome coronavirus 2 (SARS-CoV-2) entry genes across tissues revealed a possible role of CTS-L up-regulation in the regulation of SARS-CoV-2 entry into host cells (*55*). (iii) Although excessive expression and secretion of pCTS-L might be pathogenic in lethal bacterial infections, it is not yet known whether pCTS-L still occupies a protective role during nonlethal microbial infections. This is important, because appropriate pCTS-L expression might still be needed to mount sufficient inflammatory responses to fight against nonlethal viral infections (*56*). Last, the possible roles of other CTS proteins (e.g., CTS-S) in lethal sepsis were not investigated, although extracellular levels of CTS-B, CTS-L, and CTS-S were elevated in murine and human pancreatitis (*35*) and juvenile idiopathic arthritis (*57*). Despite these limitations, our discovery of pCTS-L–neutralizing mAbs capable of disrupting its interaction with TLR4 and RAGE receptors to attenuate dysregulated inflammation has suggested an exciting possibility of developing antibody strategies to fight against lethal sepsis. It will thus be important to translate these preclinical findings into clinical applications by generating humanized pCTS-L–neutralizing mAbs for further testing of therapeutic efficacy in future clinical studies.

## MATERIALS AND METHODS

### Study design

The aim of this study was to assess the pathogenic changes of plasma pCTS-L concentrations in critically ill patients with sepsis and to develop pCTS-L domain-specific mAbs to prevent septic injury and lethality in preclinical settings. For the clinical investigation, blood samples were obtained from normal healthy controls and patients with sepsis or septic shock recruited to the Northwell Health System, and their plasma pCTS-L concentrations were assessed by immunoassays. To provide a cohort of age-matched normal healthy controls, we also obtained several healthy control serum samples from the Discovery Life Science Open Access Biorepository. Sample sizes were based on statistical power calculation, and no blinding or randomization was applied for these noninterventional observations. For the preclinical study, animals were randomly assigned to different experimental groups and treated with pCTS-L–targeting pAbs or mAbs at the indicated dosing regiments. The outcomes included animal survival rates and tissue histology scores, which were collected under blinded experimental conditions, so the identity of the groups was not revealed until after the completion of the experiments. Study design and sample sizes used for each experiment were provided in the figure legends. No data, including outlier values, were excluded. Primary data are reported in data file S1. All reagent sources are listed in table S1.

### Cell culture

Murine macrophage-like RAW 264.7 cells were obtained from the American Type Culture Collection. Primary peritoneal macrophages were isolated from WT Balb/C, WT C57BL/6, or mutant C57BL/6 mice defective in either TLR4 or both TLR4 and RAGE (7 to 8 weeks, 20 to 25 g, male or female) at 3 days after intraperitoneal injection of 2 ml of thioglycolate broth (4%) as previously described (*23*, *24*, *49*). Human blood was purchased from the New York Blood Center (Long Island City, NY, USA), and human PBMCs were isolated by density gradient centrifugation through Ficoll (Ficoll-Paque PLUS) as previously described (*23*, *24*, *49*). Murine macrophages and human PBMCs were cultured in Dulbecco’s modified Eagle’s medium supplemented with 1% penicillin/streptomycin and 10% fetal bovine serum or 10% human serum. When they reached 70 to 80% confluence, adherent cells were gently washed with, and immediately cultured in, Opti-MEM I before stimulating with crude LPS (*E. coli* 0111:B4; #L4130, Sigma-Aldrich), recombinant human SAA (catalog no. 300-13, PeproTech), HMGB1, or pCTS-L. The intracellular and extracellular concentrations of pCTS-L or various other cytokines/chemokines were determined by Western blotting analysis, cytokine antibody arrays, or enzyme-linked immunosorbent assay (ELISA) as previously described (*23*, *24*, *49*).

### MALDI-TOF mass spectrometry

To identify the 40-kDa band that was induced by human SAA in macrophage-conditioned culture medium, proteins in the cell-conditioned culture medium were resolved by SDS–polyacrylamide gel electrophoresis (SDS-PAGE), and the corresponding 40-kDa band was subjected to matrix-assisted laser desorption/ionization–time-of-flight (MALDI-TOF) MS analysis as previously described (*26*, *49*). Briefly, the 40-kDa band was excised from the SDS-PAGE gel and subjected to in-gel trypsin digestion. The mass of the tryptic peptides was measured by MALDI-TOF MS and then subjected to peptide mass fingerprinting database analysis to identify the 40-kDa protein (P40).

### Cytokine antibody arrays

Murine cytokine antibody arrays (catalog no. AAM-CYT-3-8, RayBiotech Inc., Norcross, GA, USA), which simultaneously detect 62 cytokines on one membrane, were used to measure relative cytokine concentrations in macrophage-conditioned culture medium or murine serum as described previously (*23*, *49*). Human cytokine antibody C3 arrays (catalog no. AAH-CYT-3-8), which detect 42 cytokines on one membrane, were used to determine cytokine concentrations in human PBMC-conditioned culture medium or human plasma samples as previously described (*23*, *24*, *49*).

### Open Surface Plasmon Resonance

We used the Nicoya Life Science gold nanoparticle–based OpenSPR technology (Kitchener, ON, Canada) to characterize protein-protein interactions following the manufacturer’s instructions. For instance, highly purified recombinant pCTS-L, human TLR4 (ECD-His Tag, residues 1 to 631, catalog no. 10146-H08B, Sino Biological Inc.), or extracellular domain of human RAGE (residues 1 to 344, catalog no. 11629-H08H, Sino Biological Inc.) was respectively immobilized on nitrilotriacetic acid (NTA) sensor chip (catalog no. SEN-Au-100-10-NTA), and TLR4, RAGE, or pCTS-L was applied as analyte at different concentrations. To determine the binding affinities of mAbs to human or murine pCTS-L, highly purified human or murine pCTS-L was immobilized on the NTA sensor chip (catalog no. SEN-Au-100-10-NTA), and various mAbs were applied at various concentrations. The response units were recorded over time, and the binding affinity was estimated as the equilibrium dissociation constant *K*_d_ using Trace Drawer Kinetic Data Analysis v.1.6.1 (Nicoya Life Science).

### ClusPro protein-protein docking

To gain insight into specific details of receptor-ligand interactions, we used the ClusPro Web Server (https://cluspro.org) (*58*) for protein-protein docking to find structures of the pCTS-L/receptor complexes that exhibited the minimal Gibbs free energy under the assumption that conformational changes were moderate upon protein-protein interactions. Docking with each energy parameter set resulted in 10 models defined by centers of highly populated clusters of lowest Gibbs free energy docked structures. We selected the model with the lowest Gibbs free energy to predict possible structure of the pCTS-L/receptor complexes.

### Animal model of lethal endotoxemia and sepsis

This study was conducted in accordance with policies of the National Institutes of Health (NIH) *Guide for the Care and Use of Laboratory Animals* and approved by the Institutional Animal Care and Use Committee (IACUC) of the Feinstein Institutes for Medical Research (FIMR). To evaluate the role of pCTS-L in lethal sepsis, Balb/C mice (7 to 8 weeks old, 20 to 25 g, male or female) were subjected to lethal endotoxemia or sepsis by intraperitoneal administration of bacterial endotoxins (LPS, *E. coli* 0111:B4, #L4130, Sigma-Aldrich) or by a surgical procedure termed as CLP as previously described (*23*, *24*, *49*). Briefly, the cecum of Balb/C mice was ligated at 5.0 mm from the cecal tip and then punctured once with a 22-gauge needle. To alleviate surgical pain, all animals were given a dose of anesthetics (e.g., buprenorphine, 0.05 mg/kg, subcutaneously) immediately before CLP surgery, and small amounts of anesthetics (such as bupivacaine and lidocaine) were injected locally around the incision site immediately after the closure of the abdominal wound of CLP surgery. At 30 min after CLP, all animals were given a subcutaneous dose of imipenem/cilastatin (0.5 mg per mouse) (Primaxin, Merck & Co. Inc.) and resuscitation with normal sterile saline solution (20 ml/kg). Anti–pCTS-L polyclonal or monoclonal IgGs were intraperitoneally administered to septic mice at the indicated doses and time points, and animal survival rates were monitored for up to 2 weeks. Every attempt was made to limit the number of animals used in the present study according to the Animal Research: Reporting of In Vivo Experiments (ARRIVE) guidelines for reducing the number of animals in scientific research developed by the British National Centre for the Replacement, Refinement and Reduction of Animals in Research (NC3Rs). In addition, all experiments were performed in accordance with NIH policies and the *Guide for the Care and Use of Laboratory Animals* and approved by the IACUC of the FIMR (IACUC Protocol #2008-033 Term IV).

### Real-time RT-PCR analysis of Ctsl expression

Male Balb/C mice were subjected to lethal sepsis by CLP, and animals were sacrificed at 24 hours after CLP. Total RNAs were isolated from various tissues using a TRIzol reagent kit and reversely transcribed into the first-strand cDNA. Following reverse transcription, quantitative PCR was performed with TaqMan Master Mix (Thermo Fisher Scientific) for murine *Ctsb* (#4351370, assay ID Mm01310506_m1), *Ctse* (#4351370, assay ID Mm00456010_m1), *Ctsl* (#4351370, assay ID Mm00515597_m1), *Ctss* (#4351370, assay ID Mm01255859_m1), and glyceraldehyde-3-phosphate dehydrogenase (GAPDH) gene (#4351370, assay ID Mm99999915-g1) to quantify the mRNA expression levels of respective genes using an ABI 7900HT Fast Real-time PCR system. The relative abundance of various *Cts* mRNA expression in control group was normalized by GAPDH.

### Genotyping

We obtained a few breeding pairs of heterozygous *Ctsl* KO [NOD.129P2(B6)-Ctsl^tmCptr^/Rcl J] mice from The Jackson Laboratory (stock no. 008352, Bar Harbor, ME, USA) and bred heterozygous *Ctsl*^+/−^ females with heterozygous *Ctsl*^+/−^ males to produce homozygous *Ctsl* KO as well as WT littermates for the proposed animal studies. To verify the genotypes of WT and *Ctsl* KO mice, tail biopsies were digested in Direct PCR Lysis Reagent (catalog no. 102-T, Viagen Biotech Inc.) containing proteinase K (0.4 μg/ml; catalog no. EO0491, Thermo Fisher Scientific), and lysate containing genomic DNA was amplified by PCR using the following primers: WT *Ctsl*: 5′-GGAGGAGAGCGATATGGG-3′ (forward) and 5′-AGCCATTCACCACCTGCC-3′ (reverse); mutant *Ctsl*: 5′-AATTCGCCAATGACAAGACG-3′ (forward) under the following conditions: 95°C for 3 min, followed by 37 cycles of 95°C for 15 s, 60°C for 15 s, and 72°C for 15 s. The PCR products were resolved on a 2% agarose gel and visualized by ethidium bromide staining. We subjected the genetic background-, age-, and sex-matched WT littermate (*Ctsl*^+/+^) and pCTS-L–deficient (*Ctsl*^−/−^) mice to lethal sepsis and compared sepsis-induced liver injuries between WT and *Ctsl*-deficient mice at 24 hours after CLP.

### Tissue injury

The liver and intestine samples were harvested at 24 hours after CLP or pCTS-L intraperitoneal administration and fixed in 10% buffered formalin before being embedded in paraffin. Paraffin-embedded tissues were cut into 5-μm sections, stained with hematoxylin and eosin, and examined under light microscopy. As previously described (*59*), liver parenchymal injury was assessed in a blinded fashion by the sum of three different Suzuki scores ranging from 0 to 4 for sinusoidal congestion, vacuolization of hepatocyte cytoplasm, and parenchymal necrosis. Using a weighted equation with a maximum score of 100 per field, the parameter scores were calculated and then averaged as the final liver injury score in each experimental group.

### RNA-seq analysis

To elucidate the mechanisms underlying pCTS-L–mediated pathogenesis, normal healthy Balb/C mice were intraperitoneally given recombinant pCTS-L (20 mg/kg), and various tissues were harvested 24 hours later to isolate total RNA to characterize the expression profile of a full catalog of transcripts by RNA-seq (GENEWIZ, South Plainfield, NJ, USA). Gene ontology (GO) analysis and Kyoto Encyclopedia of Genes and Genomes (KEGG) pathway analysis were applied to analyze the differentially expressed genes by using String online tools (https://string-db.org/cgi/input.pl). Differential expression analysis was performed using the Wald test (DESeq2) to generate *P* values and log_2_ fold changes. Genes with an adjusted *P* value of <0.05 and absolute log fold change of >2 were defined as differentially expressed.

### Clinical characterization of septic patients

This study was approved by the Institutional Review Board (IRB) of the FIMR (IRB protocols #18-0184 and #18-0700) and endorsed by written informed consent from all participants providing blood samples. The inclusion criteria for septic patients were (i) acute diagnosis (within 24 hours) of sepsis with suspected (cultures drawn and antibiotic given) or confirmed (via culture results) infections, (ii) receiving vasopressor (norepinephrine, phenylephrine, epinephrine, dopamine, vasopressin, or angiotensin II), (iii) scheduled blood sampling, and (iv) have an indwelling arterial of central venous catheter for blood drawing. The exclusion criteria for septic patients were (i) significant underlying immunodeficiency or known autoimmune disease, (ii) recent (within 1 month) administration of any immunomodulating drugs including glucocorticoids or drugs used for treatment of autoimmune diseases, (iii) diagnosis of sepsis >24 hours before recruitment, (iv) inability to obtain consent, (v) member of a protected population (pregnant, prisoner), and (6) patients for whom parents or legal guardians did not wish to give consent.

Blood samples (5.0 ml) were collected from 8 healthy control subjects and 10 septic patients recruited to North Shore University Hospital and the Long Island Jewish Medical Center between 2018 and 2019 (listed as “P1-P10” in data file S1). Patients were diagnosed with sepsis or septic shock by the Sepsis-3 criteria (*60*), and blood samples (5.0 ml) were obtained within 24 hours of diagnosis of sepsis (defined as “time 0”), followed by two subsequent blood sampling at 24 and 72 hours after the initial diagnosis. To provide a cohort of age-matched normal healthy controls, we also purchased several healthy control serum samples from the Discovery Life Science Open Access Biorepository. Subsequently, these clinical samples were assayed for pCTS-L by Western blotting and human pCTS-L ELISA kit (catalog no. MBS7254442, MyBioSource.com) with reference to purified recombinant human pCTS-L at various dilutions.

### Statistical analysis

All data were assessed for normality by the Shapiro-Wilk test before conducting appropriate statistical tests among groups. The comparison of two independent samples was assessed by Student’s *t* test and Mann-Whitney test for Gaussian and non-Gaussian distributed samples, respectively. For comparison among multiple groups with normal data distribution, the differences were analyzed by one-way analysis of variance (ANOVA), followed by the Fisher least significant difference test. For comparison among multiple groups with nonnormal (skewed) distribution, the statistical differences were evaluated with the nonparametric Kruskal-Wallis ANOVA test. For survival studies, the Kaplan-Meier method was used to compare the differences in mortality rates between groups with the nonparametric log-rank post hoc test. The Spearman rank or the Pearson correlation coefficient test was used to evaluate associations between two quantitative variables that exhibit nonnormal or normal distribution, respectively. A *P* value of <0.05 was considered statistically significant.

## Notes

View/request a protocol for this paper from *Bio-protocol*.
