# Supplementary material for: Identification of procathepsin L (pCTS-L)–neutralizing monoclonal antibodies to treat potentially lethal sepsis
Source: Sci Adv. 2023 Feb 3;9(5):eadf4313. doi: 10.1126/sciadv.adf4313 (PMC9897667; doi:10.1126/sciadv.adf4313)
Supplement: Supplementary file 1 — Supplementary Materials and Methods Figs. S1 to S11 Tables S1 and S2 [file sciadv.adf4313_sm.pdf]

Science Advances
AAAS

Supplementary Materials for

Identification of procathepsin L (pCTS-L)–neutralizing monoclonal antibodies to treat potentially lethal sepsis

Cassie Shu Zhu *et al.*

Corresponding author: Haichao Wang, hwang@northwell.edu

Sci. Adv. **9**, eadf4313 (2023)
DOI: 10.1126/sciadv.adf4313

The PDF file includes:

Supplementary Materials and Methods
Figs. S1 to S11
Tables S1 and S2
Legends for movie S1 and S2
Legend for data file S1

Other Supplementary Material for this manuscript includes the following:

Movies S1 and S2
Data file S1

This PDF file includes:

Materials and Methods

Fig. S1. Western blotting analysis of LPS- and SAA-induced pCTS-L release and expression in human peripheral blood mononuclear cells (PBMCs).

Fig. S2. Comparison of blood pCTS-L concentrations between age-matched healthy controls and septic patients.

Fig. S3. Expression and purification of recombinant human and murine pCTS-L.

Fig. S4. Critical role of TLR4 and RAGE in pCTS-L-induced production of KC/GRO-α and MIP-2/GRO-β in peritoneal macrophages.

Fig. S5. Requirement of TLR4 and RAGE for pCTS-L-induced cytokine/chemokine production *in vivo*.

Fig. S6. Sequence of 24 synthetic peptides of human and murine pCTS-L and epitope mapping of anti-pCTS-L rabbit and murine serum.

Fig. S7. Anti-pCTS-L polyclonal IgGs (pAbs) significantly attenuated pCTS-L-induced inflammation.

Fig. S8. Epitope mapping and antigen affinities of human and murine pCTS-L-reactive monoclonal antibodies.

Fig. S9. Recombinant pCTS-L expressed in human kidney cells similarly activated human PBMCs.

Fig. S10. Top ten models of ClusPro Protein-Protein Docking of pCTS-L interaction with TLR4 or RAGE

Fig. S11. Proposed model for the pCTS-L-neutralizing mAbs against lethal sepsis.

Table S1. Reagent sources

Table S2. Demographics of two cohort of normal healthy controls and 10 septic patients

Other Supplementary Materials for this manuscript include the following:

Movie S1. Rotating image of the TLR4-pCTS-L complex with the lowest Gibbs free energy.

Movie S2. Rotating image of the RAGE-pCTS-L complex with the lowest Gibbs free energy.

Data file S1. Primary data.

Materials and Methods

Western blotting

The concentrations of pCTS-L in murine macrophage- or human PBMC-conditioned culture medium, murine serum or human plasma were determined by Western blotting analysis using commercial mAb (#C0994, Sigma-Aldrich) or pAb (Cat. # SC6498, Santa Cruz), or home-made rabbit anti-murine pCTS-L pAb. The concentrations of cellular pro-Casp-11 and mature Casp-11 in pCTS-L-stimulated peritoneal macrophages were determined by Western blotting using rabbit anti-mouse caspase-11 monoclonal antibodies (Cat. # ab180673, Abcam). Equal volume of cell-conditioned culture medium or murine/human serum were resolved on sodium dodecyl sulfate (SDS)-polyacrylamide gels and transferred to polyvinylidene difluoride (PVDF) membranes. After blocking with 5% nonfat milk, the membranes were incubated with the appropriate antibodies (anti-pCTS-L, 1:1000; anti-pro-Casp-11, 1:1000) overnight. Subsequently, the membranes were incubated with the appropriate secondary antibodies, and the immune-reactive bands were visualized by chemiluminescence. The relative levels of specific proteins were determined using the UN-SCAN-IT Gel Analysis Software Version 7.1 (Silk Scientific Inc., Orem, UT, USA) with reference to appropriate controls. The liver content of fibrinogen-γ (FGG) was measured by Western blotting analysis using mouse anti-FGG monoclonal antibody following standard procedures with reference to a house-keeping protein, β-actin.
Wild-type *Ctsl*$^{+/+}$ or *Ctsl* $^{-/-}$ KO (NOD.129P2(B6)-Ctsl^{tmCptr}/Rcl J) mice were intraperitoneally administered with bacterial endotoxin (8.0 mg/kg), and animals were sacrificed at 24 h post endotoxemia to harvest blood. The concentration of pCTS-L in murine serum was determined by Western blotting analysis using home-made rabbit anti-murine pCTS-L polyclonal antibodies. Equal volume of serum was resolved on sodium dodecyl sulfate (SDS)-polyacrylamide gels and transferred to polyvinylidene difluoride (PVDF) membranes. After blocking with 5% nonfat milk, the membranes were incubated with the appropriate antibodies (anti-pCTS-L, 1:1000) overnight. Subsequently, the membranes were incubated with the appropriate secondary antibodies, and the immune-reactive bands were visualized by chemiluminescence.

ELISAs

To confirm the relative cytokines levels, ELISA kits were used to quantitate the concentrations of pCTS-L in parallel with several biomarkers of experimental and clinical sepsis. An ELISA kit for human pCTS-L (Cat.# MBS7254442, MyBioSource.com) was used measure blood pCTS-L levels in normal healthy controls and septic patients. In addition, we obtain ELISA kits for other inflammatory biomarkers including GRO (Cat. # ELH-GRO-1, RayBiotech), IL-6 (Cat. # MBS8123859, MyBioSource), IL-8 (Cat. #ELH-IL8, RayBiotech), MCP-1 (Cat. #MBS7721397), and HMGB1 (Cat.# OKCD03560, Aviva Systems Biology) to measure their levels in septic patients as well as in gender-matched normal healthy controls.

To measure extracellular SQSTM1 in macrophage-conditioned culture medium, an ELISA kit was obtained from the Novus Biological Inc. (Cat. # NBP2-61300). Peritoneal macrophages were isolated from wild-type C57BL/6 mice (male, 8-10 Weeks, 20-25 g) or mutant mice deficient in both TLR4 and RAGE at 3d post intraperitoneal administration of thioglycolate

broth, and stimulated with recombinant murine pCTS-L for 6 h or 24 h in serum-free DMEM medium. Macrophage-conditioned culture medium were collected, and extracellular levels of KC and MIP-2 were measured by using mouse KC/CXCL1 Douset ELISA Kit (Cat.# DY453-05, R&D System) and mouse MIP-2/CXCL2 Q Douset ELISA Kit (Cat. # DY452-05, R&D Systems).

Preparation of recombinant human and murine pCTS-L proteins
The cDNA encoding for human (residue 17-333) and murine (residue 18-334) pCTS-L was cloned into a pReceiver expression vector downstream of a T7 promoter with an N-histidine tag, and recombinant pCTS-L protein was expressed in *E. coli* BL21 (DE3) pLysS as previously described (*49*). The inclusion body-associated recombinant pCTS-L protein was isolated by differential centrifugation and urea solubilization before refolding in Tris buffer (pH 8.0) containing N-lauroylsarcosine. The recombinant pCTS-L protein with N- His Tag was then further purified by histidine-affinity chromatography, followed by extensive Triton X-114 extractions to remove contaminating endotoxins. Recombinant pCTS-L protein was tested for LPS content by the chromogenic *Limulus* amebocyte lysate assay (Endochrome; Charles River), and the endotoxin content was less than 0.01 U per microgram of recombinant protein. For comparison, we also obtained bacterial products-free pCTS-L expressed in human HEK293 cells (Cat. #. CT1-H5222, Acro Biosystems) as an additional control for recombinant protein expressed in *E. coli*.

Generation of anti-pCTS-L polyclonal and monoclonal antibodies
Polyclonal antibodies were generated in female New Zealand white rabbits by the Covance Inc. (Princeton, NJ, USA) using recombinant murine and human pCTS-L in combination with Freund's complete adjuvant following standard procedures. Blood samples were collected in 3-week cycles of immunization and bleeding, and the antibody titers were determined by direct pCTS-L ELISA. Total IgGs and pCTS-L antigen-binding IgGs were purified from anti-pCTS-L rabbit serum using Protein A and pCTS-L-affinity chromatography as described in the Supplemental Materials.

The monoclonal antibodies were generated in Balb/C and C57BL/6 mice by the GenScript (Piscataway, NJ, USA) using highly purified human or murine pCTS-L following standard procedures. Blood samples were collected every two weeks, and serum titers were assessed by indirect ELISA and Western blotting analysis. After four immunizations, mouse splenocytes were harvested, fused with mouse Sp2/0 myeloma cell line, and screened for antibody-producing hybridomas by indirect ELISA, dot blotting, and Western blotting analysis. After limiting dilution, purified hybridoma clones were generated to produce mAbs following standard procedures.

Affinity purification of polyclonal antibodies
Total IgGs and pCTS-L antigen-binding IgGs were purified from anti-pCTS-L rabbit serum using Protein A and pCTS-L-affinity column chromatography, respectively. Briefly, rabbit serum was pre-buffered with PBS and slowly loaded onto the Protein A/G Sepharose (Cat. # ab193262) column to allow sufficient binding of IgGs. After washing with 1xPBS to remove unbound serum components, the IgGs were eluted with acidic buffer (0.1 M glycine-HCl, pH 2.8), and then immediately dialyzed into 1×PBS buffer at 4°C overnight. For pCTS-L antigen-

affinity purification, recombinant murine pCTS-L was conjugated to cyanogen bromide (CNBr)-activated Sepharose4 agarose beads (Cat. # 17098101, GE Healthcare), and the pCTS-L-conjugated Sepharose beads were then loaded onto columns. Following repetitive washings with acid buffer (0.1 M Acetic/Sodium Acetate, 0.5 M NaCl, pH4.0) and alkali buffer (0.1 M Tris-HCl, 0.5 NaCl, pH 8.0), anti-pCTS-L total IgGs were slowly loaded onto the column, and the flow-through fractions were collected. Following repetitive washing with 1×PBS buffer, the pCTS-L-binding antibodies were eluted with acidic elution buffer, and immediately neutralized in 1×PBS buffer.

Peptide dot blotting

A library of 24 synthetic peptides corresponding to different regions of human or murine pCTS-L sequence were synthesized at the Genscript, and spotted (0. 1 μg in 2.5 μl) onto nitrocellulose membrane (Thermo Scientific, Cat No. 88013). Subsequently, the membrane was probed with anti-pCTS-L rabbit or murine serum, or IgGs isolated from anti-pCTS-L rabbit serum or murine hybridoma cultures following a standard protocol.

Supplemental Figures

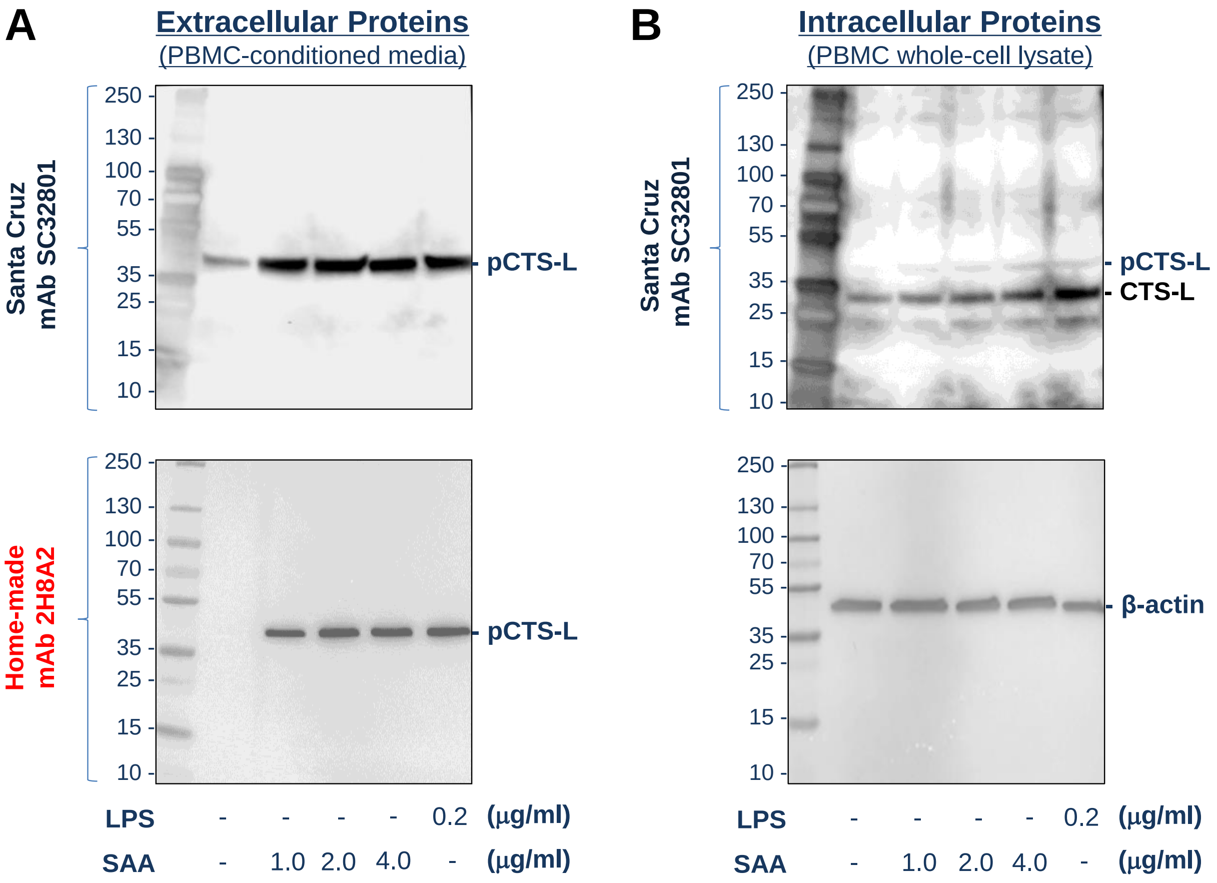

Fig. S1. Western blotting analysis of LPS- and SAA-induced pCTS-L release and expression in human peripheral blood mononuclear cells (PBMCs).

Human PBMCs were stimulated with LPS or SAA at indicated concentrations for 16 hours, and proteins in the PBMC-conditioned culture medium (**Panel A**) or whole-cell lysate (**Panel B**) were immunoblotted with different anti-CTS-L monoclonal antibodies. For cellular proteins, a house-keeping protein, β-actin was used to confirm equivalent loading of different samples. In the culture medium conditioned by LPS- or SAA-stimulated human PBMCs, two different mAbs similarly recognized a single band of 40 kDa, confirming that LPS and SAA induced pCTS-L release in human PBMCs. In PBMC whole-cell lysate, the Santa Cruz mAb SC32801 not only recognized a 40-kDa band that was up-regulated by SAA or LPS stimulation, but also a 25-35 kDa band that matched the molecular weight of mature CTS-L.

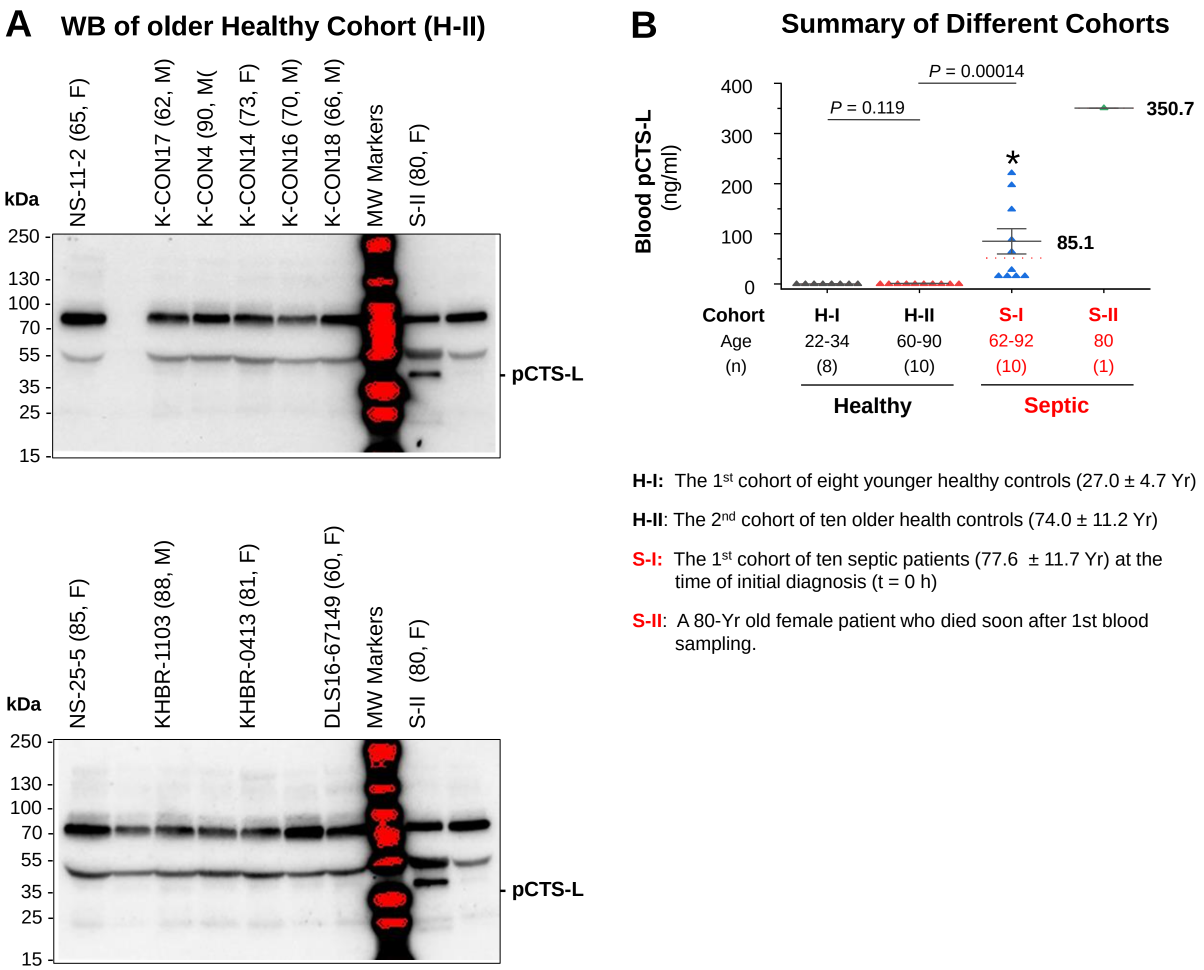

Fig. S2. Western blotting analysis of serum pCTS-L in a cohort of older healthy controls.

A) Western blotting analysis of serum pCTS-L in a cohort of older healthy controls. Serum of ten older healthy controls and a septic patient (S-II) who soon died after blood sampling was assayed for pCTS-L content by Western blotting using a murine anti-human pCTS-L monoclonal antibody. Note that pCTS-L was markedly elevated in the blood of a septic patient, but barely detected in most healthy controls.

B) Comparison of blood pCTS-L content between different cohorts of healthy controls and septic patients. Blood concentrations of pCTS-L in different cohorts of healthy controls (H-I and H-II) and septic patients (S-I) were measured by ELISA. Note the basal blood pCTS-L concentrations were not significantly different between two healthy cohorts, but were significantly elevated in a cohort of age-matched septic patients (S-I, ten septic patients at t = 0).

A

Amino acid sequence of human and murine precathepsin L

```
Signal Sequence                Propeptide Inhibitor Domain
1      10        20        30        40        50        60        70        80        90        100
MNLLLLLAVLCLGTALATPKFDQTFSAEWHQWKSTHRRLYGTNEEEWRRAIWEKNMRIIQLHNGEYSNGQHGFSMEMNAFGDMTNEEFRQVVNGYRHQKH
||:.|:||:.|||.|.||:.||.:..|:|.:||..|.||||.|||.||||:|||||::|:|||.||..|:|.|:|.||||||||:||||||:||::::|.
MNPTLILAAFCLGIASATLTFDHSLEAQWTKWKAMHNRLYGMNEEGWRRAVWEKNMKMIELHNQEYREGKHSFTMAMNAFGDMTSEEFRQVMNGFQNRKP

         110       120       130       140       150       160       170       180       190       200
KKGRLFQEPLMLKIPKSVDWREKGCVTPVKNQGQCGSCWAFSASGCLEGQMFLKTGKLISLSEQNLVDCSHAQGNQGCNGGLMDFAFQYIKENGGLDSEE
:||::|||||...:|:||||||||.||||||||||||||||||||:|.||||||.:||:||||||||||||||.:|||:||||||||||:||||:::||||||||
RKGKVFQEPLFYEAPRSVDWREKGYVTPVKNQGQCGSCWAFSATGALEGQMFRKTGRLISLSEQNLVDCSGPQGNEGCNGGLMDYAFQYVQDNGGLDSEE

         210       220       230       240       250       260       270       280       290       300
SYPYEAKDGSCKYRAEFAVANDTGFVDIPQQEKALMKAVATVGPISVAMDASHPSLQFYSSGIYYEPNCSSKNLDHGVLLVGYGYEGTDSNKNKYWLVKN
|||||||:.||||...:.||||||||||||:|||||||||||||||||||:||.|.|..||..|||:||:||||.::|||||:||||:|.|:|:.||||||||
SYPYEATEESCKYNPKYSVANDTGFVDIPKQEKALMKAVATVGPISVAIDAGHESFLFYKEGIYFEPDCSSEDMDHGVLVVGYGFESTESDNNKYWLVKN

         310       320        330
SWGSEWGMEGYIKIAKDRDNHCGLATAASYPVVN        (Murine preCTS-L)
|||.||||.||:|:||||.|||||:|:||||||.|
SWGEEWGMGGYVKMAKDRRNHCGIASAASYPTV         (Human preCTS-L)
```

Structural domains of human and murine precathepsin L

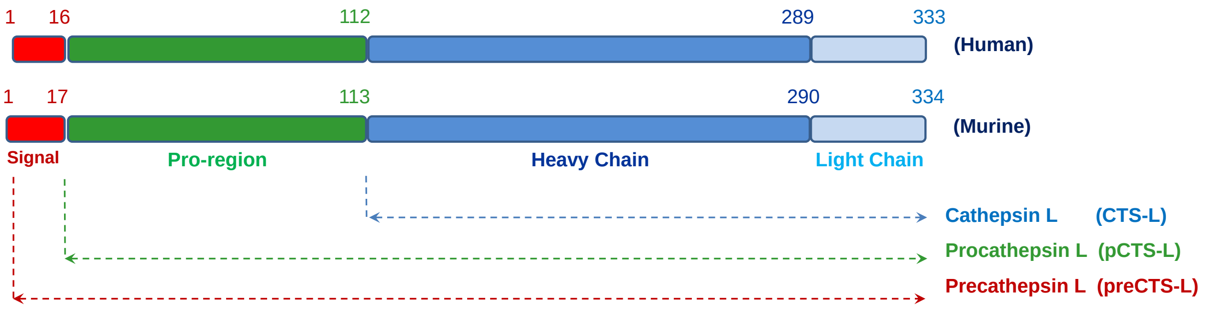

Homology (%)

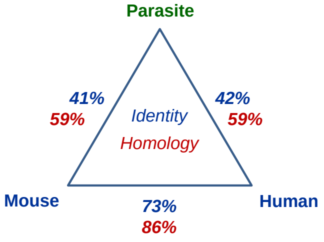

B SDS-PAGE

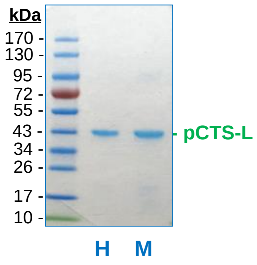

Fig. S3. ***Expression and purification of recombinant human and murine procathepsin L (pCTS-L).***

A) Amino acid sequence, structural domains, and homology between human and murine precathepsin L (preCTS-L). Human and murine <u>pre</u>-<u>c</u>a<u>t</u>hep<u>s</u>in <u>L</u> (**pre-CTS-L**) are synthesized as a 333 or 334 amino acid precursor with an N-terminal 16- or 17-residue leader **signal** sequence (shown in red), which can be removed in the <u>ER</u> to release the procathepsin L (pCTS-L). The

pCTS-L is folded with the assistance of three disulfide bonds (not shown) and the pro-region (green), which can then be cleaved in the endosome to release the cathepsin L (CTS-L). Upon delivery to the lysosome, the CTS-L is further proteolytically processed to produce the active enzyme consisting of a heavy (dark blue) and light chain (light blue). The amino acid sequence homology among mouse, human, and a parasite (liver fluke, Fasciola hepatica) CTS-L is also shown.

B) Expression and purification of recombinant human and murine pCTS-L proteins. Recombinant human (“H”) and murine (“M”) pCTS-L corresponding to residue 17-333 or 18-334 of respective precathepsin L with a N-histidine tag were expressed in *E. coli* BL21 (DE3) pLysS cells as insoluble inclusion bodies. After sonication to disrupt the bacteria, the inclusion bodies were isolated by differential centrifugation following extensive washing in 1 × PBS containing 1% Triton X-100. The inclusion bodies were then solubilized in 8 M urea, and refolded by dialysis in 10 mM Tris buffer (pH 8.0) containing N-lauroylsarcosine. Subsequently, the recombinant proteins were subjected to extensive Triton X-114 extractions to remove contaminating endotoxins.

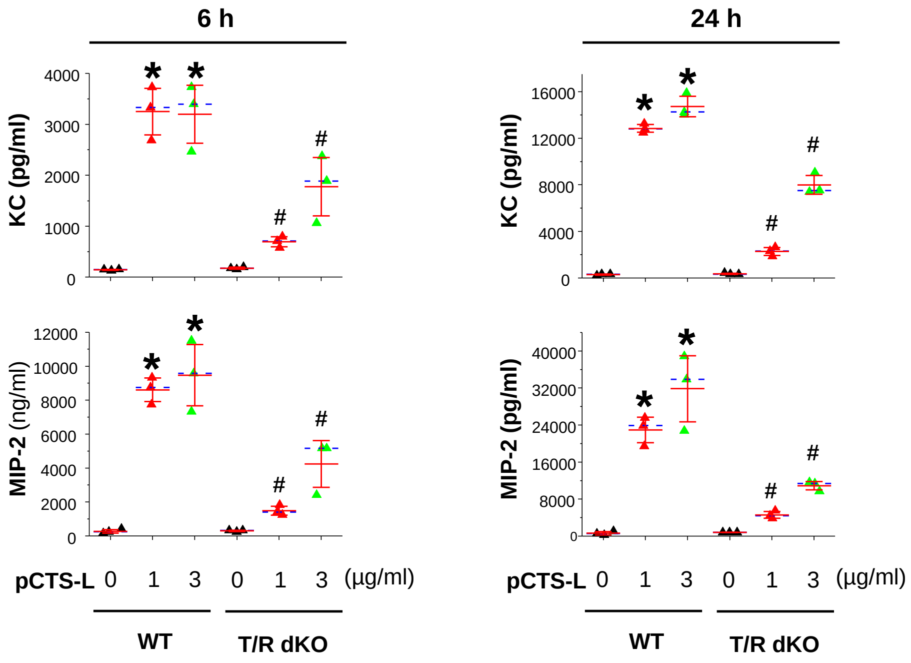

Fig. S4. ***Critical role of TLR4 and RAGE in pCTS-L-induced production of KC/RGO-α and MIP-2/GRO-β in peritoneal macrophages*.**

Thioglycollate-elicited primary macrophages were isolated from wild-type (WT) C57BL/6 or mutant C57BL/6 mice deficient in both TLR4 and RAGE (T/R dKO). Following stimulation with pCTS-L (1.0 or 3.0 μg/ml) for 6 or 24 h, the extracellular levels of MIP-2 and KC were determined by ELISA assays and expressed as mean ± SEM of three experiments (n = 3). *, $P < 0.05$ versus "- pCTS-L" negative control of respective genotype (WT or T/R dKO); #, P < 0.05 versus WT " + PCTS-L" positive control treated with pCTS-L at the same concentrations (1.0 or 3.0 μg/ml). Note that the disruption of both TLR4 and RAGE completely abrogated pCTS-L (1.0 μg/ml)-induced production of both MIP-2 (homolog of human GRO-β) and KC (homolog of human GRO-α) at both 6 and 24 h post pCTS-L stimulation.

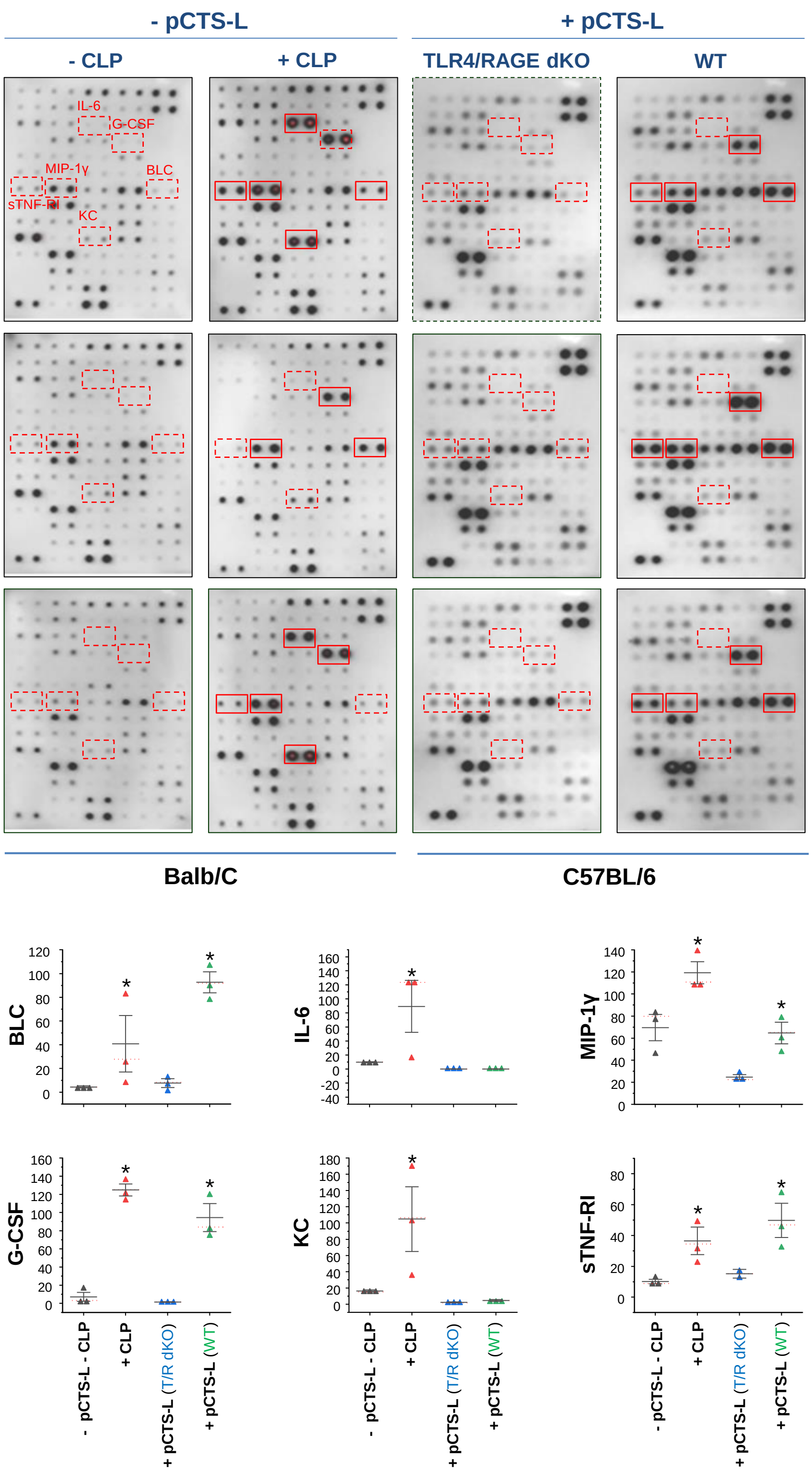

Fig. S5. ***Requirement of TLR4 and RAGE for pCTS-L-induced cytokine/chemokine production in vivo.***

Wildtype C57BL/6 (WT) or mutant C57BL/6 mice deficient in both TLR4 and RAGE (T/R dKO) were intraperitoneally administered with recombinant pCTS-L (40 mg/kg), and animals were euthanized to harvest blood to measure serum levels of 62 cytokines by Cytokine Antibody Arrays. The relative cytokine levels were expressed as mean ±SEM of three animals (n = 3) in arbitrary units. *, $P < 0.05$ versus "- pCTS-L" negative control or "+ pCTS-L" positive control of TLR4/RAGE double KO mice (T/R dKO). Note that pCTS-L significantly elevated blood levels of BLC, G-CSF, MIP-1γ and sTNF-RI in the wildtype, but not in mutant mice deficient in TLR4 and RAGE, confirming an important role for these receptors in pCTS-L-mediated dysregulated inflammation. For comparison, serum samples from normal healthy Balb/C mice or Balb/C mice subjected to CLP surgery were used as additional controls. Note that CLP surgery induced a marked elevation of blood concentrations of three cytokines/chemokines (i.e., BLC, G-CSF, MIP-1γ) and sTNF-RI in the WT, but not in T/R dKO, some of which (i.e., IL-6 and KC) were known as surrogate markers of experimental sepsis.

A Sequence of murine (M) and human (H) pCTS-L peptides.

Peptide #	Murine pCTS-L	Peptide #	Human pCTS-L
1	18-TPKFDQTFSAEWHQWKS-34	1	18-TLTFDHSLEAQWTKWKAM-35
2	26-SAEWHQWKSTHRRLYGTN-43	2	26-EAQWTKWKAMHNRLYGMN-43
3	36-HRRLYGTNEEEWRRA-50	3	36-HNRLYGMNEEGWRRA-50
4	46-EWRRAIWEKNMRMIQLHNGEYS-67	4	46-GWRRAVWEKNMKMIELHNQEY-66
5	60-MIQLHNGEYSNGQHG-74	5	83-MTSEEFRQVM-92
6	71-HGFSMEMNAFGD-82	6	69-GKHSFTMAMNAFGD-82
7	93-NGYRHQKHKKGRLFQEP-109	7	93-NGFQNRKPRKGKVFQEP-109
8	106-FQEPLMLKIPKSVDWREK-123	8	106-FQEPLFYEAPRSVDWREK-123
9	121-REKGCVTPVKNQGQCGS-137	9	124-GYVTPVKNQGQCGS-137
10	137-SCWAFSASGCLEGQMFLKT-155	10	137-SCWAFSATGALEGQMFRKT-155
11	156-GKLISLSEQNLVDCSHAQG-174	11	156-GRLISLSEQNLVDCSGPQG-174
**12**	175-**NQGCNGGLMDFAFQYIKEN**-193	**12**	175-**NEGCNGGLMDYAFQYVQDN**-193
**13**	194-**GGLDSEESYPYEAKDGSCKYR**-214	**13**	194-**GGLDSEESYPYEATEESCKYN**-214
14	203-PYEAKDGSCKYRAEFAVA-220	14	203-PYEATEESCKYNPKYSVA-220
15	221-NDTGFVDIPQQ-231	15	221-NDTGFVDIPKQ-231
16	228-IPQQEKALMKAVATVGP-244	16	228-IPKQEKALMKAVATVGP-244
17	243-GPISVAMDASHPSLQFYS-260	17	242-VGPISVAIDAGHESFL-257
18	257-QFYSSGIYYEPNCSSKNLD-275	18	258-FYKEGIYFEPDCSSEDMD-275
19	274-LDHGVLLVGYGYEGTDSNKN-293	19	274-MDHGVLVVGYGFESTESDNN-293
20	292-KNKYWLVKNSWGSEW-306	20	292-NNKYWLVKNSWGEEW-306
21	306-WGMEGYIKIAKDRDN-320	21	307-GMGGYVKMAKDRRN-321
22	316-KDRDNHCGLATAASYPVVN-334	22	316-KDRRNHCGIASAASYPTV-333
23	120-WREKGCVTPVKNQG-133	23	120-WREKGYVTPVKNQG-133
24	250-DASHPSLQFYS-260	24	250-DAGHESFLFYK-260

B Epitope mapping of rabbit and mouse anti-pCTS-L serum.

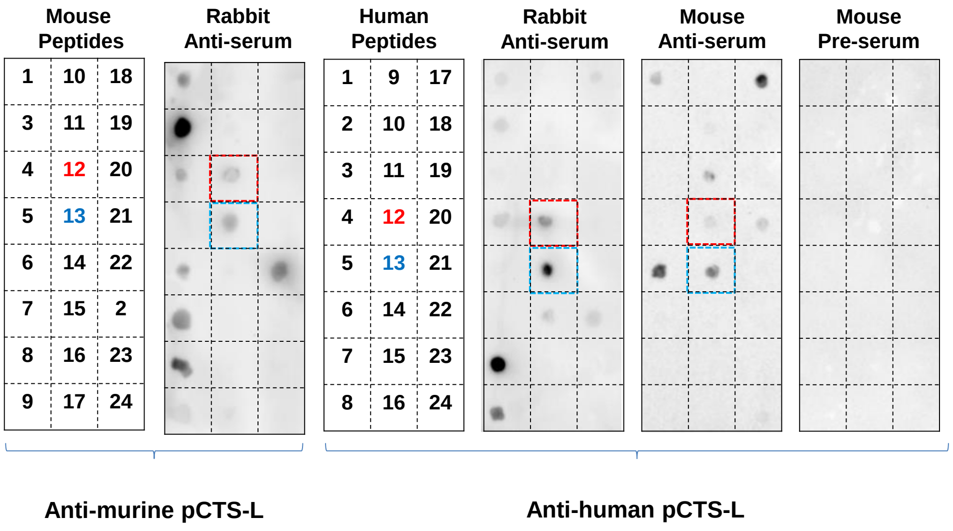

Fig. S6. Sequence of 24 synthetic peptides of human and murine pCTS-L and epitope mapping of anti-pCTS-L rabbit and murine serum.

A) Sequence of 24 peptides corresponding to different regions of murine and human pCTS-L

B) Epitope mapping of rabbit or murine serum raised against murine or human pCTS-L.

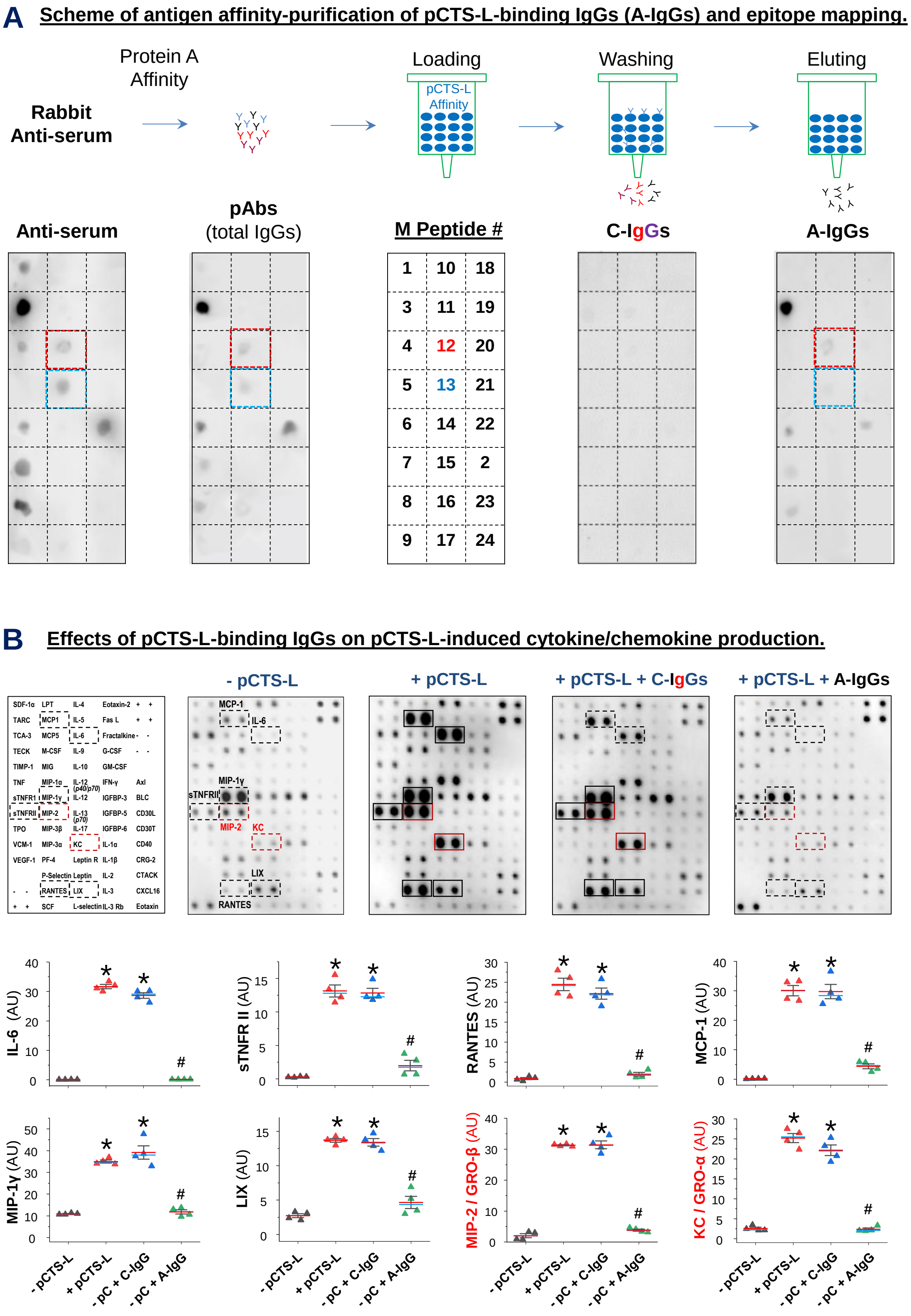

Fig. S7. Anti-pCTS-L polyclonal IgGs (pAbs) significantly attenuated pCTS-L-induced inflammation.

A) Scheme for purifying pCTS-L antigen-binding IgGs from rabbit total IgGs. Total IgGs were purified from anti-murine pCTS-L rabbit serum using Protein A affinity chromatography, and pCTS-L-binding IgGs were then purified by pCTS-L antigen-affinity chromatography. The non-pCTS-L-binding control immunoglobulins ("C-IgG") were collected in the washout fractions before the pCTS-L antigen-bound antibodies ("A-IgGs") were eluted from the column by an acidic buffer into solution with physiological pH to prevent acid-catalyzed antibody denaturation.

B) pCTS-L antigen-affinity purified IgGs abrogated the pCTS-L-induced cytokines and chemokines. Thioglycollate-elicited peritoneal macrophages were isolated from Balb/C mice, and stimulated with recombinant pCTS-L either alone or in the presence of control IgGs (C-IgGs) or antigen-affinity purified IgGs ("A-IgGs") for 16 h, and the extracellular levels of 62 different cytokines and chemokines were measured by Cytokine Antibody Arrays. The relative cytokine levels were expressed as mean ±SEM of two experiments in duplicates (n = 4) in arbitrary units. *, $P < 0.05$ versus "- pCTS-L" negative control; #, $P < 0.05$ versus "+ pCTS-L" positive control. Note that antigen-affinity purified IgGs effectively abrogated the pCTS-L-induced secretion of IL-6, sTNF RII and six different chemokines such as RANTES, MCP-1, MIP-1γ, LIX, MIP-2 and KC.

A **Epitope mapping of mAbs raised against murine pCTS-L (Mp).**

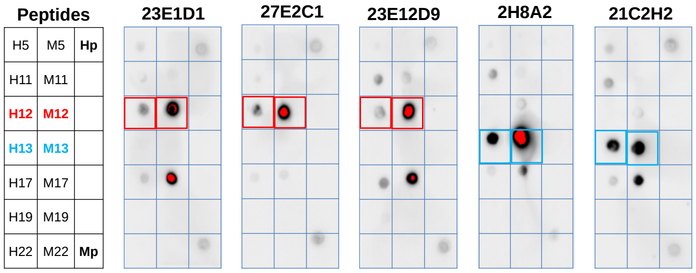

Isotype	IgG1	IgG2b	IgG2b	IgG2a	IgG2b
Epitope	P12	P12	P12	P13	P13
K_D for Mp (nM)	ND	ND	ND	20.3 ± 9.5	ND
K_D for Hp (nM)	ND	ND	ND	15.1 ± 10.9	ND
CLP Protection	N	N	N	Y	-

B **Epitope mapping of mAbs raised against human pCTS-L (Hp).**

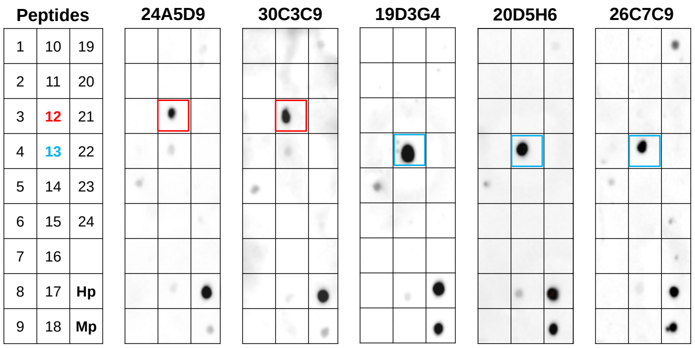

Isotype	IgG2b	IgG2b	IgG2a	IgG2b	IgG2b
Epitope	P12	P12	P13	P13	P13
K_D for Mp (nM)	ND	467.0	ND	11.2 ± 6.9	3.6 ± 1.5
K_D for Hp (nM)	20.2	2.0	4.5	10.1 ± 1.6	2.9 ± 0.6
CLP Protection	N	N	N	Y	Y

Fig. S8. Epitope mapping and antigen affinities of human and murine pCTS-L-reactive monoclonal antibodies.

A) Use of eight pairs of homologous peptides corresponding to different regions of murine (M) human (H) pCTS-L to determine the epitope profile of monoclonal antibodies raised against murine pCTS-L. The isotype, relative binding affinity (K_D) to human pCTS-L (Hp) or murine pCTS-L (Mp), as well as protective efficacy in CLP sepsis were also noted.

B) Use of 24 peptides corresponding to different region of human pCTS-L to characterize the epitope profile of monoclonal antibodies raised against human pCTS-L. Note that three P13-reactive mAbs recognized both recombinant human pCTS-L (Hp) and murine pCTS-L (Mp) on the dot blots.

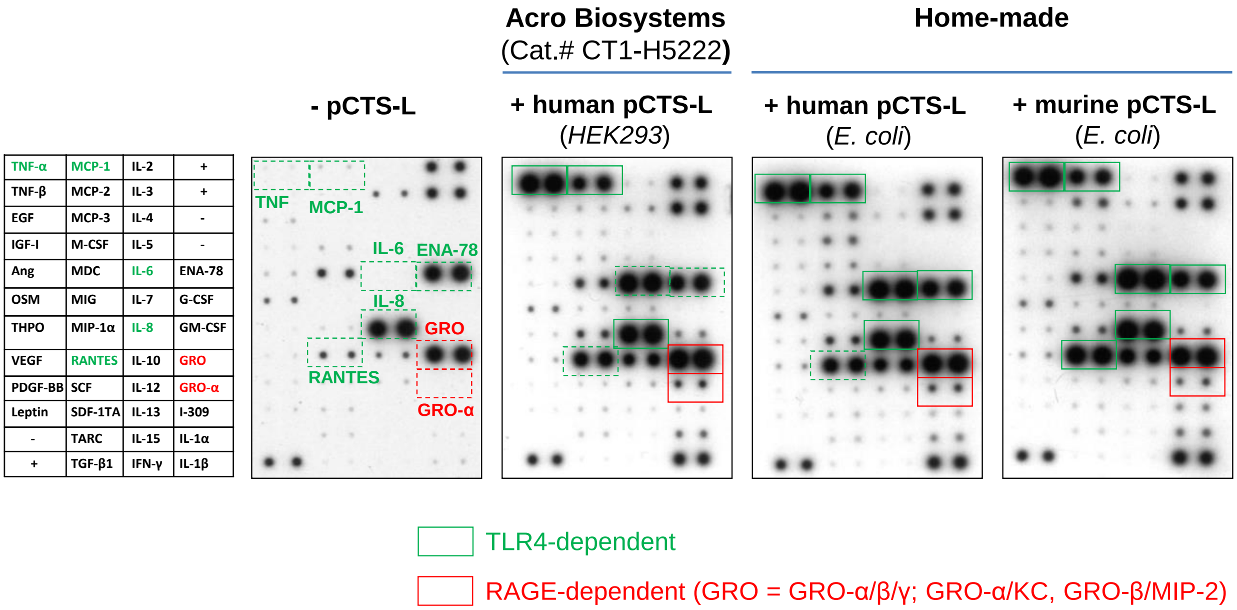

Fig. S9. ***Recombinant pCTS-L expressed in human kidney cells similarly activated human PBMCs.***

Human PBMCs were isolated from blood of normal healthy subjects, and stimulated with recombinant pCTS-L expressed either in human HEK293 kidney cell line or *E. coli* for 16 h, and extracellular levels of various cytokines and chemokines were measured by Cytokine Antibody Arrays.

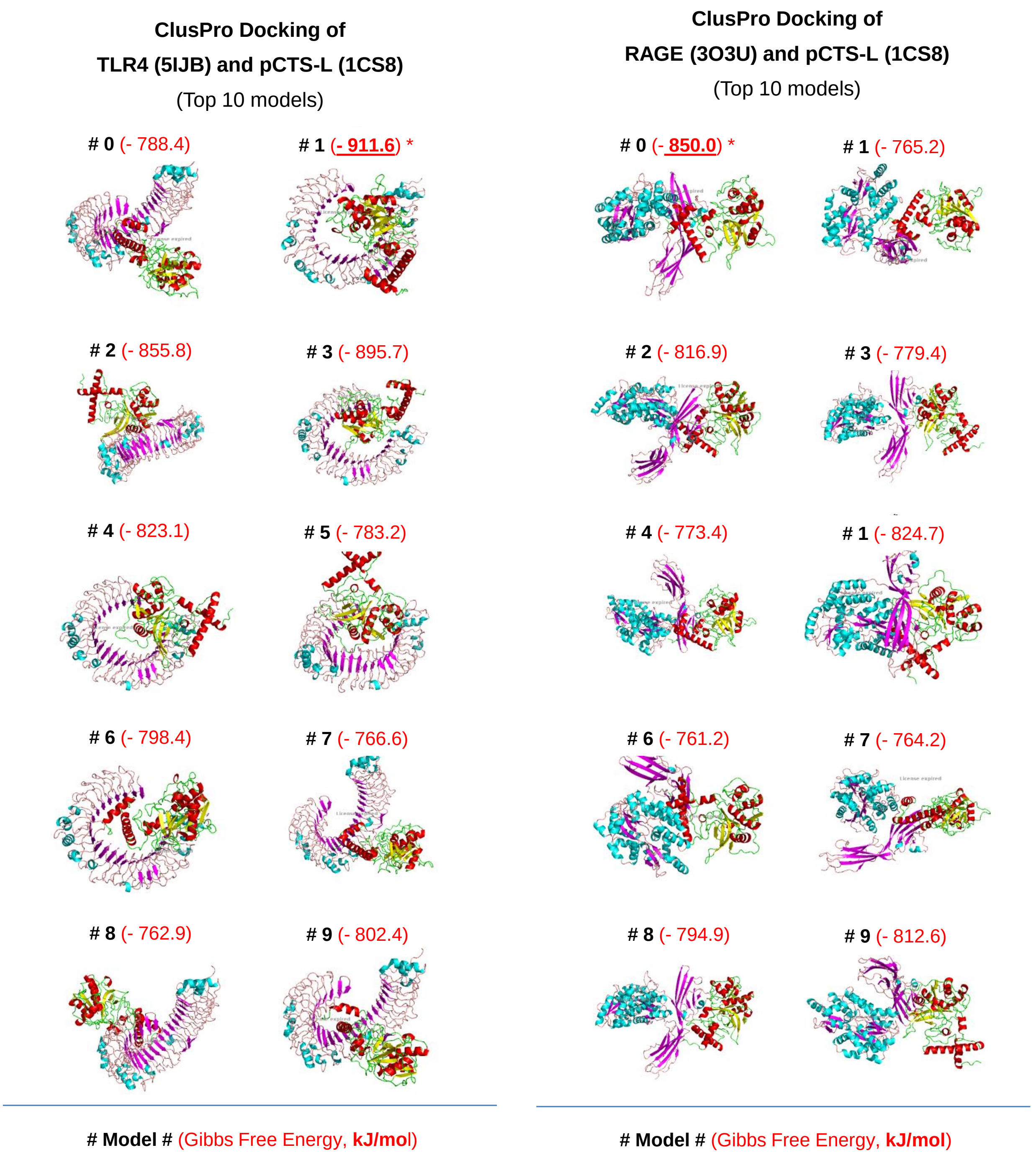

Fig. S10. Top ten models of ClusPro Protein-Protein Docking of pCTS-L interaction with TLR4 or RAGE.

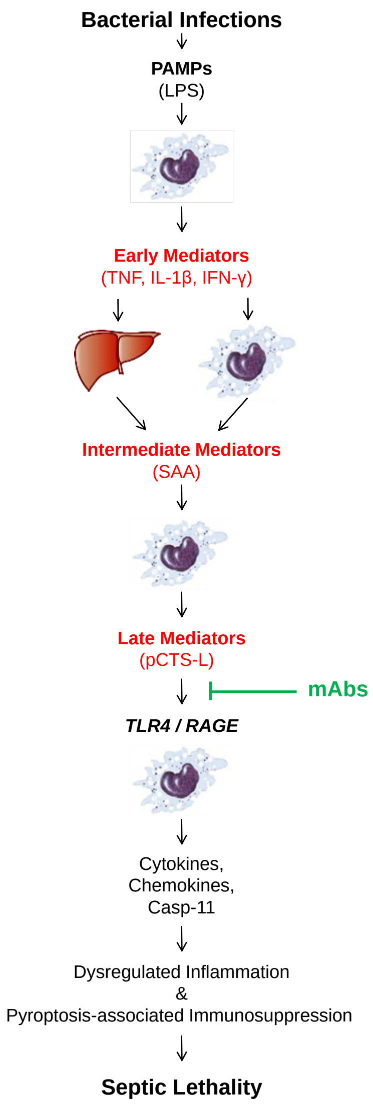

Fig. S11. Proposed model for pCTS-L-neutralizing mAbs protection against lethal sepsis.

Pathogen-associated molecular pattern molecules (PAMPs such as bacterial lipopolysaccharide, LPS) relies on cell surface pattern recognition receptors (PRR, TLR4) to activate innate immune cells to immediately release "early" proinflammatory mediators (such as TNF, IL-1β, and IFN-γ), which then stimulate hepatocytes and innate immune cells to synthesize and secrete a proinflammatory mediator, serum amyloid A (SAA). SAA then activate innate immune cells to upregulate and secrete procathepsin-L (pCTS-L), which binds to cell surface PRRs such as TLR4 and RAGE to induce: **i**) the expression of cytokines/chemokines to trigger dysregulated inflammation; and **ii**) the expression of pro-Casp-11 to activate inflammasome and pyroptosis. The pCTS-L-mediated dysregulation inflammation and pyroptosis-associated immunosuppression may adversely contribute to the pathogenesis of lethal sepsis. A panel of P13-reactive mAbs, such as mAb20, could bind to pCTS-L to interrupt its interaction with TLR4 and RAGE, thereby impairing pCTS-L-mediated dysregulated inflammation to confer protection against lethal sepsis.

Supplemental tables

Table S1. Reagent sources

REAGENTS	SOURCE	IDENTIFIER
**Antibodies**		
Mouse anti-β-actin antibody	Sigma-Aldrich	Cat. # A1978
Mouse anti-human procathepsin L monoclonal antibody	Sigma-Aldrich	Cat.# C0994
Goat anti-human procathepsin L (pCTS-L) polyclonal antibodies	Santa Cruz Biotechnology	Cat. # sc-6498
Mouse anti-human cathepsin L monoclonal antibody	Santa Cruz Biotechnology	Cat. # sc-32801
Rabbit anti-mouse caspase-11 monoclonal antibody	Abcam	Cat. # ab180673
Mouse anti-fibrinogen γ monoclonal antibody	Santa Cruz	Cat. # sc-133226
HRP conjugated donkey anti-rabbit IgG	GE Healthcare	Cat. # NA934
**Chemicals, Peptides, and Recombinant Proteins**		
Crude bacterial endotoxin (lipopolysaccharide, LPS)	Sigma-Aldrich	*E. coli 0111:B4*
Human serum	Sigma-Aldrich	Cat. # H3667
Recombinant human SAA (also termed Apo-SAA	PeproTech	Cat. # 300-13
Recombinant human procathepsin L (pCTS-L)	Acro Biosystems	Cat.# CT-H5222
Cyanogen bromide (CNBr)-activated Sepharose4 agarose beads	GE Healthcare	Cat. # 17098101
Protein A/G Sepharose®	Abcam	Cat. # ab193262
Dulbecco's modified Eagle medium (DMEM)	Invitrogen/Life Technologies	Cat. # 11995-065
OPTI-MEM I Reduced-Serum Medium	ThermoFisher Scientific	Cat. # 31985062
Penicillin/streptomycin	Invitrogen/Life Technologies	Cat. # 15140-122
**Cytokine Antibody Arrays, ELISA Kits, and SPR Sensor Chip**		
Human procathepsin L (pCTS-L) ELISA kit	MyBioSource	Cat. # MBS7254442
Human IL-6 ELISA Kit	MyBioSource	Cat. # MBS8123859
Human IL-8 ELISA Kit	RayBiotech	Cat. #ELH-IL8
Human monocyte chemotactic protein 1 (MCP-1) ELISA Kit	MyBioSource	Cat. # MBS7721397
Human CXCL1 (GRO-α) ELISA Kit	RayBiotech	Cat. # ELH-GROα-1

Human GRO-α/β/γ ELISA Kit	RayBiotech	Cat. # ELH-GRO-1
Human HMGB1 ELISA Kit	Aviva Systems Biology	Cat.# OKCD03560
Mouse CXCL1/KC DouSet ELISA	R & D Systems	Cat. # DY453-05
Mouse CXCL2/MIP-2 Douset ELISA	R & D Systems	Cat. # DY452-05
Human, Mouse, Rat p62/SQSTM1 ELISA Kit	Novus Biological Inc.	Cat. # NBP2-61300
**NTA sensor chip**	Nicoya Lifesciences	Cat. # SEN-Au-100-10-NTA
Murine Cytokine Antibody Arrays	RayBiotech Inc	Cat. #. AAM-CYT-3-8
Human Cytokine Antibody C3 Arrays	RayBiotech Inc	Cat. # AAH-CYT-3-8

Mice

Balb/C mice	Jackson Laboratory	Stock # 000651
Heterozygous Ctsl KO (NOD.129P2(B6)-Ctsltm1Cptr/RclJ) mice	Jackson Laboratory	Stock # 008352

Software and Algorithms

UN-SCAN-IT Gel Analysis Software Version 7.1	Silk Scientific Inc.	
ClusPro Protein-Protein docking	https://cluspro.org	

Table S2. Demographics of two cohorts of normal healthy controls and one cohort of septic patients.

		Healthy Control I (H-I)	Healthy Control II (H-II)	Septic Patients (S-I)
**Sample Size (n)**		8	10	10
**Age**	**Range**	22 – 34	60-90	62 - 94
	**Mean ± SD**	27.0 ± 4.7	74.0 ± 11.2 ^{ns}	77.6 ± 11.7 ^{ns}
**Gender ratio (M/F)**		4/4	5/5	5/5
**[pCTS-L]** (ng/ml) (Mean ± SEM)		0.14 ± 0.05	0.9 ± 0.43*	85.1 ± 25.1*

Note: ns, P = 0.50 for the mean age between Healthy Control II (H-II) and Septic Patients (S-I).
*, P = 0.00014 for mean blood pCTS-L concentration between age-matched Healthy Control II (H-II) and Septic Patients (S-I).

Other Supplementary Materials for this manuscript include the following:

Movie S1. Rotating image of the TLR4-pCTS-L complex with the lowest Gibbs free energy.

Movie S2. Rotating image of the RAGE-pCTS-L complex with the lowest Gibbs free energy.

Data file S1. Primary data.
